# VASC: Dimension Reduction and Visualization of Single-cell RNA-seq Data by Deep Variational Autoencoder

**DOI:** 10.1016/j.gpb.2018.08.003

**Published:** 2018-12-18

**Authors:** Dongfang Wang, Jin Gu

**Affiliations:** MOE Key Laboratory of Bioinformatics, BNRIST Bioinformatics Division & Center for Synthetic and Systems Biology, Department of Automation, Tsinghua University, Beijing 100084, China

**Keywords:** Single cell RNA sequencing, Deep variational autoencoder, Dimension reduction, Visualization, Dropout

## Abstract

Single-cell RNA sequencing (scRNA-seq) is a powerful technique to analyze the transcriptomic heterogeneities at the single cell level. It is an important step for studying cell sub-populations and lineages, with an effective low-dimensional representation and **visualization** of the original scRNA-Seq data. At the single cell level, the transcriptional fluctuations are much larger than the average of a cell population, and the low amount of RNA transcripts will increase the rate of technical **dropout** events. Therefore, scRNA-seq data are much noisier than traditional bulk RNA-seq data. In this study, we proposed the **deep variational autoencoder** for scRNA-seq data (VASC), a deep multi-layer generative model, for the unsupervised **dimension reduction** and visualization of scRNA-seq data. VASC can explicitly model the dropout events and find the nonlinear hierarchical feature representations of the original data. Tested on over 20 datasets, VASC shows superior performances in most cases and exhibits broader dataset compatibility compared to four state-of-the-art dimension reduction and visualization methods. In addition, VASC provides better representations for very rare cell populations in the 2D visualization. As a case study, VASC successfully re-establishes the cell dynamics in pre-implantation embryos and identifies several candidate marker genes associated with early embryo development. Moreover, VASC also performs well on a 10× Genomics dataset with more cells and higher dropout rate.

## Introduction

Characterizing the cellular states at the single cell level is crucial for understanding the cell–cell heterogeneities and the biological mechanisms that cannot be observed in the average behaviors of a bulk of cells. Single-cell RNA sequencing (scRNA-seq) is a promising high-throughput technique to simultaneously profile the transcriptomes of a large number of individual cells [1]. Thousands of genes are simultaneously expressed in a single cell. Expression levels of these genes are usually tightly regulated in regard to a limited number of cellular states. Finding an effective low-dimensional representation of the scRNA-seq data is the basic step for the data visualization and the downstream analysis, such as the cell lineage establishment and the cell sub-population identification [2]. Currently, several traditional dimension reduction methods used for the bulk RNA-seq data analysis, such as principal components analysis (PCA) [3] and t-distributed stochastic neighbor embedding (t-SNE) [4], are still widely used for the scRNA-seq data analysis. However, the transcriptional burst effects and low amounts of RNA transcripts in single cells make the scRNA-seq data much noisier than the bulk RNA-seq data. For example, the scRNA-seq data have many unexpected dropout events (many data points are zero or near-zero) [5]. These noises make those traditional methods inefficient. To improve the analysis, one useful strategy is to explicitly mimic the data generation process by a probabilistic model. For example, the zero-inflated factor analysis (ZIFA), which combines the probabilistic factor analysis with conditional dropout probability, was developed to find the latent low dimension subspace [6]. However, ZIFA can only model linear patterns by a single hidden layer, which limits its performance on the datasets with complex cellular states in the original data space. Another strategy is to embed the cells into another low-dimensional space by preserving the cell–cell similarity (or distance) in the original data space. But, this kind of methods, such as single-cell interpretation via multiple kernel learning (SIMLR) [7], frequently change the basic topological information in the embedded space.

In recent years, deep probabilistic hidden models have shown superior performances in representing complex features of high-dimensional data, especially for images and speeches [8], [9]. In this study, we developed a deep model, deep variational autoencoder for scRNA-seq data (VASC), to analyze and visualize the scRNA-seq data. VASC can capture non-linear variations and automatically learn a hierarchical representation of the input data. In addition, it uses the Gumbel distribution to better model the zero and near-zero dropout events. We systematically compared VASC with several state-of-the-art dimension reduction methods on 20 datasets. Results show that VASC has superior performance in most cases and exhibits a broader dataset compatibility.

## Methods

### VASC: the method overview

VASC, a generative model based on the deep variational autoencoder (VAE) [9], [10], [11], was designed to find an effective low-dimensional representation and facilitate the visualization of scRNA-seq datasets. It modeled the distribution of high-dimensional original data P(***X***), by a set of latent variables ***z*** (the dimension of ***z*** should be much lower than ***X***, in particular, being two for visualization). The primary goal of VASC is to find the optimal ***z*** capturing the intrinsic information of the input data. In a probabilistic view, the posterior distribution P(***z***|***X***) could be treated as the best distribution of ***z*** given the observed data ***X***. However, P(***z***|***X***) is usually intractable. Variational inference is thus proposed to solve this problem by designing another common distribution family Q(***z***|***X***) (also known as variational distribution) to approximate P(***z***|***X***). The minimization of the Kullback–Leibler (KL) divergence between the two distributions is usually adopted for the approximation. The variational distribution Q(***z***|***X***) should be sufficiently representative to model the complex information of P(***z***|***X***) in the scRNA-seq datasets, and on the other hand, should be tractable for efficient computation. In VASC, deep neural networks were used to explicitly model the variational distribution. Unlike the traditional variational inference methods, deep neural networks can approximate arbitrary functions and can be optimized efficiently using the stochastic gradient descent methods.

**Figure 1:**
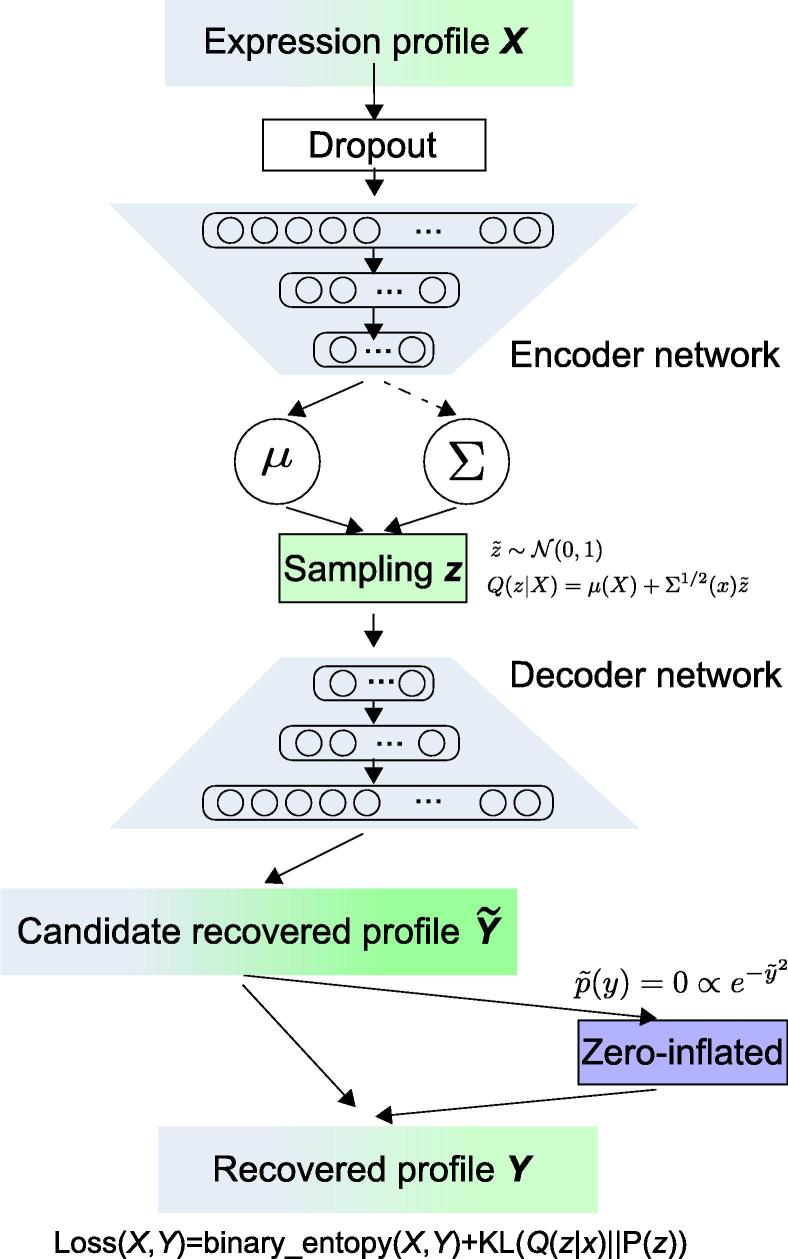
**Overview of VASC workflow** VASC consists of three parts: the encoder network, the decoder network, and the zero-inflated layer. Both the encoder and decoder networks are designed as three-layer fully-connected neural networks. VASC, variational autoencoder for scRNA-seq data; X, input expression profile for one cell; μandΣ, mean and covariance of the latent Gaussian distribution; z, samples from the latent Gaussian distribution; $\tilde{Y}$, recovered expression profile by the decoder network; Y, recovered expression profile after zero inflation; KL, Kullback–Leibler divergence; Q(*z|X*), variational distribution; P(*z*), prior standard normal distribution. Loss(X,Y) indicates the binary entropy between original profile and recovered profile plus the KL divergence between variational distribution and prior distribution.

Generally, VASC has three major parts, namely, the encoder network, the decoder network, and the zero-inflated (ZI) layer (Figure 1). The encoder network, designed as a three-layer neural network, generates the parameters of the variational distribution. It should be noted that before the first layer, we added a “dropout” noise layer [12], which randomly set some data points in the original expression matrix as zero. From a computational point of view, it introduced additional random noises for the sample training, which can reduce the overfitting risk during the learning process. We assumed a multi-dimensional Gaussian distribution for Q(***z***|***X***) of latent variables ***z*** given the expression values ***X***, of which mean and variance parameters could be generated by the encoder network. Then, the learned Q(***z***|***X***) was used to re-generate pseudo samples ***X***’ by the decoder network, another three-layer neural network. Finally, a ZI layer, based on a double-exponential distribution, was designed to mimic the dropout events by randomly setting some data points as zero [6], [13]. The Gumbel distribution instead of the conditional binomial distribution was used in the ZI layer for the back-propagation [14], [15]. VASC was optimized by a stochastic gradient descent-based RMSprop methods [16], aimed to minimize an auxiliary loss function of the KL divergence between Q(***z***|***X***) and P(***z***|***X***). After the auto-encoding procedure, a 2D representation was learned for visualization and other downstream analysis.

### Datasets

To demonstrate the performance of VASC, we analyzed 22 scRNA-seq datasets (Table 1). The first 20 datasets were obtained from the Hemberg group (https://hemberg-lab.github.io/scRNA.seq.datasets/), with ‘scater’ toolkit [34] used for quality control. The human pre-implantation embryo dataset (Petropoulus) [32] with detailed annotations was obtained via ArrayExpress (https://www.ebi.ac.uk/arrayexpress/; accession No. E-MTAB-3929). The PBMC3k dataset was downloaded from 10× Genomics (https://support.10xgenomics.com/single-cell-gene-expression/datasets).

**Table 1:** The list of scRNA-seq datasets used in this study

**Dataset No.**	**Dataset name**		**No. of cells**	**No. of genes**	**Protocol**	**No. of reads**	**No. of cell types**	**Ref.**
1	Baron	Human-1	1937	20,125	inDrop	Around 6000	14	[17]
2		Human-2	1724					
3		Human-3	3605					
4		Human-4	1303					
5		Mouse-1	822	14,878			13	
6		Mouse-2	1064					

7	Biase		56	25,733	SMARTer	37.9 million	4	[18]

8	Camp		777	19,020	SMARTer	1–5 million	7	[19]

9	Darmanis		466	22,088	SMARTer	2,838,000	9	[20]

10	Deng		268	22,431	Smart-Seq Smart-Seq2	1–70 million	6	[21]

11	Goolam		124	41,427	Smart-Seq2	1–10 million	5	[22]

12	Klein		2717	24,175	inDrop	208,000	4	[23]

13	Kolodziejczyk		704	38,615	SMARTer	9 million	9	[24]

14	Li		561	55,186	SMARTer	-	9	[25]

15	Patel		430	5948	Smart-Seq	-	5	[26]

16	Pollen		301	23,730	SMARTer	∼50,000	11	[27]

17	Usoskin		622	25,334	STRT-Seq	1.14 million	11	[28]

18	Xin		1600	39,851	SMARTer	∼0.95 million	8	[29]

19	Yan		90	20,214	Tang	35.3 million	6	[30]

20	Zeisel		3005	19,972	STRT-Seq	500,000	9	[31]

21	Petropoulos		1529	19,651	Smart-Seq2	-	7	[32]

22	PBMC3k		2700	32,738	10X	∼2000 UMIs	8	[33]

*Note*: All protocols and reads were extracted from the original publications. UMI, unique molecular identifier.

### VAE

VASC is a deep VAE-based generative model and is designed for the visualization and low-dimensional representation of the scRNA-seq data. VAE aims to model the distribution P(***X***) of data points in a high-dimensional space *χ*, with the aid of low-dimensional latent variables ***z***. The whole model is divided into two procedures, that is, (1) generating the samples of ***z*** in the latent low-dimensional subspace, and (2) mapping them to the original space *χ*. The critical point is to generate ***z*** having the high probability to recover the observed data matrix ***X***. In this way, the generated ***z*** may be possible to capture the intrinsic information of the original data. The best choice to generate ***z***, in theory, is the posterior P(***z|X***), which however, is usually too complicated and intractable. VAE tries to use a variational probability Q(***z|X***) to approximate the posterior, by minimizing the Kullback–Leibler (KL) divergence (*D*) between Q(***z|X***) and P(***z|X***):

(1) $$D[Q\left(z|X\right)|\text{|}P\left(z\text{|}X\right)\text{]}=E_{z∼Q}\left[\log Q\left(z\text{|}X\right)-\log P\left(z|X\right)\right]$$

By applying the Bayes rule and rearranging the order, it can be re-written as:

(2) $$\log P\left(X\right)-D[Q\left(z|X\right)|\text{|}P\left(z\text{|}X\right)\text{]}=E_{z∼Q}\left[\log P\left(X\text{|}z\right)\right]-D[Q\left(z|X\right)||P\left(z\right)]$$

where P(***X***) is a constant and $E_{z∼Q}$ represents expectation over z that is sampled from Q. Therefore, minimizing the KL divergence is equivalent to maximizing the right-hand part of Equation (2). The right-hand part has a natural autoencoder structure, with the encoder Q**(*z|X***) from ***X*** to ***z*** and the decoder P**(*X|z*)** from ***z*** to ***X***. Two deep fully-connected neural networks can be used to model these two parts.

### VASC method

The whole VASC structure is shown in Figure 1. The model designs and the learning algorithms are described in detail as below.

#### Input layer

VASC uses the expression matrix from scRNA-seq data as inputs. The whole expression matrix of the transcriptome was fed directly to the model with no gene filter applied. The data were log-transformed to make the results more robust. The most important transformation, however, was to re-scale the expression of every gene in any single cell in the range [0,1] by dividing the maximum expression value of an individual gene from the same cell.

#### Dropout layer

A dropout layer [12] was added immediately after the input layer, with the dropout rate set as 0.5, which is larger than the usual choice in deep models for input layers. This layer set some features to zeros during the encoding phase, to increase the performance in model learning [35]. This layer should be a good choice for scRNA-seq data because it may be regarded as artificial and additional “dropout” events, and forces subsequent layers to learn to avoid dropout noises.

#### Encoder network

The encoder network was designed as a three-layer fully-connected neural network with decreasing dimensions 512, 128, and 32. The first layer did not use non-linear activation, which acted as an embedded PCA transformation. Many complex algorithms, including t-SNE, benefit from the PCA transformation. L1-norm regularization was added for the weights in this layer, which penalized the sparsity of the model. The next two layers were accompanied by ReLU activation, which made the output sparse and stable for deep models [36].

#### Latent sampling layer

Latent variables ***z*** were modeled by a Gaussian distribution, with the standard normal prior N(0,***I***). The encoder network was used to estimate its posterior parameters. Usually, both the parameters μandΣ needed to be estimated, with a linear activation used to estimate μ. According to our experiments, it is better to fix Σ and set logΣ=I, if the dataset only has small sample size. For the datasets with large sample size (more than 1000 cells), Σ can also be trained by the encoder network. A ‘softplus’ activation was used for the estimation of logΣ. Since the neural network does not have a stochastic layer and thus could not be tackled by back-propagation algorithm, a re-parameterization trick was used to remove the randomness in input data. It is easy to see, drawing a sample ***z*** from $N\left(\mu ,\Sigma \right)$ is equivalent to drawing a sample $\tilde{z}$ from $N\left(0,I\right)$ and then let $z=\mu +\;\Sigma^{\frac{1}{2}}\tilde{z}$ (see Section 1 of File S1 for more details).

#### Decoder network

The decoder network used the generated ***z*** to recover the original expression matrix, which was designed as a three-layer fully-connected neural network with dimensions of hidden units 32, 128, and 512, respectively, and an output layer. The first three layers used ‘ReLU’ activations and the final layer with sigmoid to make the output within [0,1] (this is why the [0,1] re-scaling transformation must be applied in the input layer).

#### ZI layer

An additional ZI layer was added after the decoder network. Adapted from the model used by ZIFA [6], we modeled the dropout events by the probability $e^{-{\tilde{y}}^{2}}$, where $\tilde{y}$ is the recovered expression value by the decoder network. Back-propagation, as mentioned before, cannot deal with stochastic units; moreover, it cannot deal with discrete units either. A Gumbel-softmax distribution [15] was thus introduced to overcome these difficulties. Suppose p is the probability for dropout and q=1-p, the sample *s* from Gumbel-softmax distribution was obtained by:

(3) $$s=\frac{\exp\left(\frac{\log p+g_{0}}{\tau }\;\right)}{\exp\left(\frac{\log p+g_{0}}{\tau }\right)+\exp\left(\frac{\log q+g_{1}}{\tau }\;\right)}\;$$

where $g_{0},g_{1}$ were sampled from a Gumbel (0,1) distribution. The samples could then be obtained by first drawing an auxiliary sample $u∼\mathrm{Uniform}\left(0,1\right)$ and then computing $g=-\log\left(-\log u\right)$. As the hyper-parameter τ→0, the generated samples from the Gumbel-softmax distribution should be identical to the samples from the Bernoulli distribution. In practice, too small values of τ makes the gradient of the whole network too small and the optimization algorithm cannot work. Our experiments showed that it would be better by setting τ between 0.5–1 for the datasets of small sample size. For the datasets with more cells, an annealing strategy may yield better results (see Section 1 of File S1 for details).

#### Loss function

The loss function as shown in the Equation (2) is composed of two components. The first part, because of the scale of our data, [0,1], was computed by binary cross-entropy loss function. The second part, controlling the divergence between posterior distribution and the prior $N\left(0,I\right)$, could be computed analytically (see Section 1 of File S1 for more details).

#### Optimization

The whole structure, now, could be optimized end-to-end using the stochastic gradient descent-based optimization algorithm. We chose the RMSprop method [16] for VASC. In addition, we set the learning rate as 0.0001, to ensure the convergence on all the datasets tested. The training processes were stopped if the training loss did not show obvious decrease within 50 epochs.

Source codes implemented by keras (https://github.com/fchollet/keras) can be found at https://github.com/wang-research/VASC.

### Benchmarking

For each dataset, we considered four state-of-the-art dimension reduction methods – PCA [3], t-SNE [4], ZIFA [6], and SIMLR [7]. For all the methods, no gene filtering was used and the same log-2 transformation was applied. For PCA and t-SNE, we used the built-in python sklearn package functions. For the datasets with more than 500 cells, we firstly applied a PCA transformation with 500 dimensions before t-SNE. Perplexity, the key parameter of t-SNE, was set as 0.2 times the number of cells as suggested previously [32]. For ZIFA, we downloaded the package and used the block_ZIFA module provided by Pierson and Yau [6], due to the large number of genes evaluated. For SIMLR, we used the R package described by Wang and colleagues [7]. For benchmarking the dimension reduction performance, *k*-means was used to obtain the predicted cell types based on their 2D representations (see Section 2 of File S1 for more details).

### Performance assessment

To measure the quality of visualization and low-dimensional representation, *k*-means clustering was applied to the 2D representations of all the aforementioned methods. Then the obtained clustering results were compared with the known cell types provided in the original references. The number of clusters, *k*, was set to number of known cell types. Four measures were used to assess the performances, including normalized mutual information (NMI) [37], adjusted rand index (ARI) [38], homogeneity [39], and completeness [39].

#### NMI

Suppose P is the predicted clustering results, and T is the known cell types (the same below), we denote the entropy of P and T as H(P) and H(T), respectively, and the mutual information between them as MI(P,T). NMI is computed as:

(4) $$\mathrm{NMI}\left(P,T\right)=\frac{\mathrm{MI}\left(P,T\right)}{\sqrt{H\left(P\right)H\left(T\right)}}$$

#### ARI

Suppose *n* is the total number of samples, $a_{i}$ is the number of samples appearing in the *i*-th cluster of *P*, $b_{j}$ is the number of samples appearing in the *j*-th types of *T*, and $n_{\mathrm{ij}}$ is the number of overlaps between the *i*-th cluster of *P* and the *j*-th type and T. ARI is computed as:

(5) $$\text{ARI}=\frac{\sum_{\mathrm{ij}}\left(\begin{array}{c} n_{\mathrm{ij}}\\ 2 \end{array}\right)-\frac{\left[\sum_{i}\left(\begin{array}{c} a_{i}\\ 2 \end{array}\right)\sum_{j}\left(\begin{array}{c} b_{j}\\ 2 \end{array}\right)\right]}{\left(\begin{array}{c} n\\ 2 \end{array}\right)}}{\frac{1}{2}\left[\sum_{i}\left(\begin{array}{c} a_{i}\\ 2 \end{array}\right)+\sum_{j}\left(\begin{array}{c} b_{j}\\ 2 \end{array}\right)\right]-\frac{\left[\sum_{i}\left(\begin{array}{c} a_{i}\\ 2 \end{array}\right)\sum_{j}\left(\begin{array}{c} b_{j}\\ 2 \end{array}\right)\right]}{\left(\begin{array}{c} n\\ 2 \end{array}\right)}}$$

#### Homogeneity

The measure homogeneity expects that every cluster only contains samples from one cell type. Suppose *H*(*T*|*P*) is the cross-entropy of cell types given the cluster *P*, the homogeneity score (*h*) is computed by:

(6) $$h=1-\frac{H(T|P)}{H\left(T\right)}\;$$

#### Completeness

The measure completeness (c) expects that samples from one cell type are assigned to the same cluster, and is computed as:

(7) $$c=1-\frac{H(P|T)}{H\left(P\right)}\;$$

For all the measures including NMI, ARI, homogeneity, and completeness, larger values (up to 1) mean better performances.

### Analysis of the PBMC3k dataset

We filtered cells with less than three detected genes (UMIs > 3). Number of UMI counts was transformed to transcript-per-million (TPM)-like values by normalizing each cell through dividing total UMI counts and then multiplying by 10,000. Log2 transformation was applied after adding a pseudo-count 1 to obtain the gene expression matrix. Due to the serious dropout events present in this dataset, gene selection is used to reduce noises. We adopted the same procedure as previously reported [40], with 1158 genes that remained. VASC was then tested on this pre-processed gene expression matrix.

## Results

### Visualization and performance comparison

**Figure 2:**
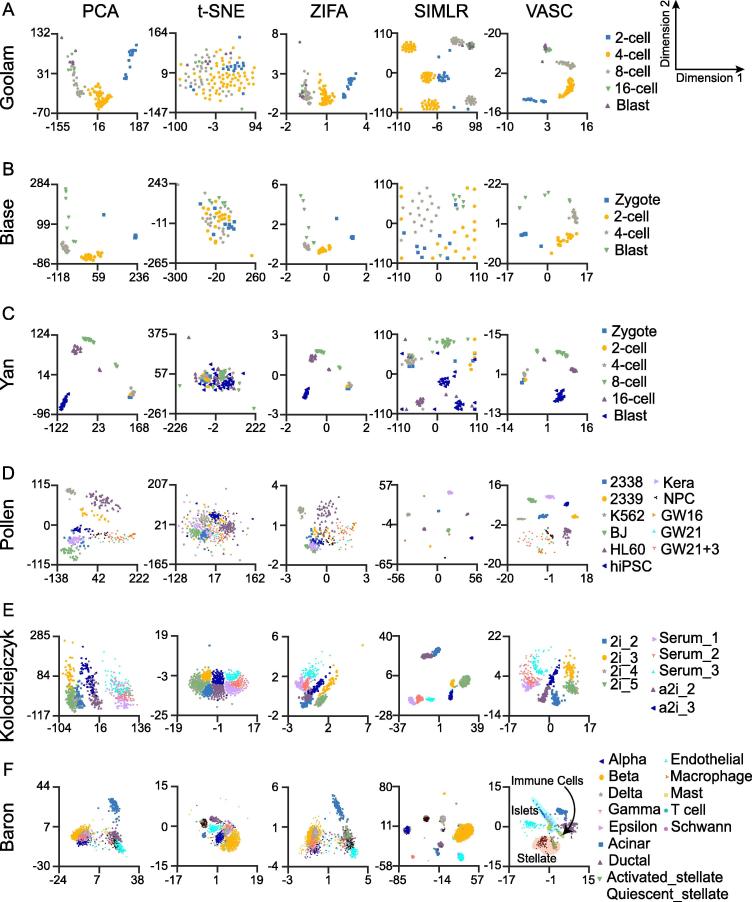
**Visualization of scRNA-seq datasets using different methods** Each data point represents a cell. Different cell types are indicated in different colors and shapes. All datasets were run by PCA, t-SNE, ZIFA, SIMLR, and VASC respectively. Cell type information was retrieved from original studies. Shown in the figures are clustering output from the Goolam [22] (**A**), Biase [18] (**B**), Yan [30] (**C**), Pollen [27] (**D**), Kolodziejczyk [24] (**E**), and Baron_human-1 [17] (**F**) datasets. Visualization of other datasets is provided in the Section 4 of File S1. PCA, principal components analysis; t-SNE, t-distributed stochastic neighbor embedding; ZIFA, zero-inflated factor analysis; SIMLR, single-cell interpretation via multiple kernel learning.

We tested the visualization performance of VASC together with four state-of-the-art dimension reduction methods, including PCA [3], t-SNE [4], ZIFA [6], and SIMLR [7], on 20 datasets with different number of cells included and sequencing protocols used (the top 20 datasets in Table 1). Firstly, we compared the 2D visualizations on six “golden” datasets (these datasets provide highly-confident cell labels), with the number of cells ranging from tens to thousands (Figure 2). Datasets reported by Goolam et al. [22], Biase et al. [18], and Yan et al. [30], respectively, were generated from studies on the embryonic development from zygote to blast cells. PCA, ZIFA, and VASC roughly re-established the developmental stages of different cell types (cells are expected to be arranged in the order of zygote, 2-cell, 4-cell, 8-cell, 16-cell, and blast cells) (Figure 2A–C). However, t-SNE and SIMLR, both of which use neighbor-preserving embedding, showed poor performance on these datasets. In contrast, VASC further separated 16-cell and blast from 8-cell stages in the Goolam dataset. Moreover, compared to PCA and ZIFA, VASC better separated blast cells from 4-cell stages, and identified one zygote as a possible outlier in the Biase dataset, whereas 4-cell stage was better separated from zygote and 2-cell stages using VASC in the Yan dataset (Figure 2A–C). These results indicate that VASC can better model the embryo developmental progression than PCA and ZIFA.

Eleven different cell types were sequenced in the fourth dataset reported by Pollen and colleagues [27]. In this case, PCA and ZIFA showed poor performance in classification (Figure 2D). In the SIMLR visualization, eleven compact clusters of cells were formed, but at least four clusters were composed of more than one cell type (the points from different cell types were stacked together for possible misleading visualization). This result was undesirable because the cells from different types should not compactly cluster together. Instead, Using VASC, eight compact clusters of cells were formed, each from the same cell type. The remaining three cell types, GW16, GW21, and GW21 + 3 (originally sampled from the germinal zone of human cortex at gestational week 16, 21, and cultured for another three weeks, respectively), were distributed in a more decentralized manner than the others. These cells, along with neural progenitor cells (NPCs), are all neural cells. Therefore, it seems reasonable that they are presented more closely using VASC.

Kolodziejczyk et al. generated a dataset when examining embryonic stem cells grown under three different conditions: serum, 2i, and alternative 2i (a2i) [24]. Moreover, there existed different experimental batches for every condition. As shown in Figure 2E, PCA separated the cells grown under the three different conditions but almost mixed the batches; ZIFA better separated the cells under different growth conditions and from different batches but incorrectly mixed one 2i cell batch (2i_2) with a2i cells; SIMLR separated most cell populations under different growth conditions and from different batches (except two batches of 2i cells), but incorrectly grouped the cells from 2i and a2i conditions. Only t-SNE and VASC separated the most cell populations, while preserving their proper relative positions.

The dataset reported by Baron et al. [17] included several sequencing subsets from four human donors and two mice. Visualization of the first donor with 1937 cells from 14 different cell types is shown in Figure 2F. On this dataset, PCA and ZIFA separated only few cell types, whereas both t-SNE and SIMLR showed better separation, although SIMLR produced more compact clusters. However, the putative clusters grouped using SIMLR contained mixtures of different cell types at various levels (for example, two kinds of stellate cells were completely mixed). Notably, VASC showed better separation of the different cell types. Furthermore, the cells from close cell lineages were clustered together. For instance, the alpha, beta, delta, gamma, and epsilon cells that are all within islets were grouped close to each other; beta cells, despite with the largest number (872 cells), were most compactly clustered by VASC. In addition, three types of immune cells, including macrophages (14 cells), mast (8 cells), and T_cells (2 cells), were grouped close to each other, whereas the Schwann cells (only 5 cells) were well separated (see the purple dots in the central region).

**Figure 3:**
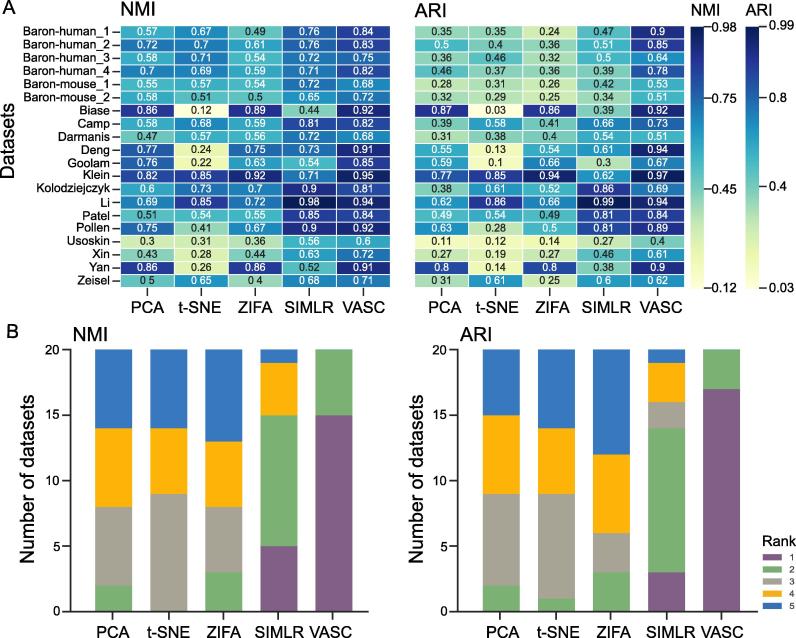
**Performance comparison using different methods** **A.** The NMI and ARI values for each method on each dataset. Clustering was performed on 2-D representations of different algorithms and then the output was compared with true cell type labels for the 20 datasets indicated. Detailed dataset information is listed in Table 1. **B.** The statistics of the ranks of the compared methods based on NMI and ARI values. For each dataset, NMI and ARI values given by different algorithms were ranked in the descending order, with rank 1 indicative the highest NMI or ARI values. The number of ranks achieved by these algorithms in the 20 datasets is then counted for distribution. NMI, normalized mutual information; ARI, adjusted rand index.

Next, to quantitatively assessing the performance of these methods in dimension reduction and visualization, we compared the cell sub-populations in the reduced subspaces (the sub-populations were identified by *k*-means clustering [41]) with the true cell type labels annotated in the original publications. Four different parameters were used, including normalized NMI [37], ARI [38], homogeneity [27], and completeness [39], to quantitatively assess the clustering performances. PCA, t-SNE, ZIFA, SIMLR, and VASC were used to systematically analyze 20 datasets, including Camp [19], Darmanis [20], Deng [21], Klein [23], Li [25], Patel [26], Usokin [28], Xin [29], Zeisel [31], besides the aforementioned databases. These comparisons showed that VASC outperformed the other methods in terms of NMI and ARI in most cases (best performances achieved on 15 and 17 out of the 20 datasets, respectively) (Figure 3A). Furthermore, VASC always ranked in the top two methods on all the tested datasets (Figure 3B) in terms of NMI and ARI, respectively. This suggests that VASC has broad compatibility with various kinds of scRNA-seq datasets (see the detailed results in the Section 4 of File S1).

### Analysis of the model stability and parameter setting

**Figure 4:**
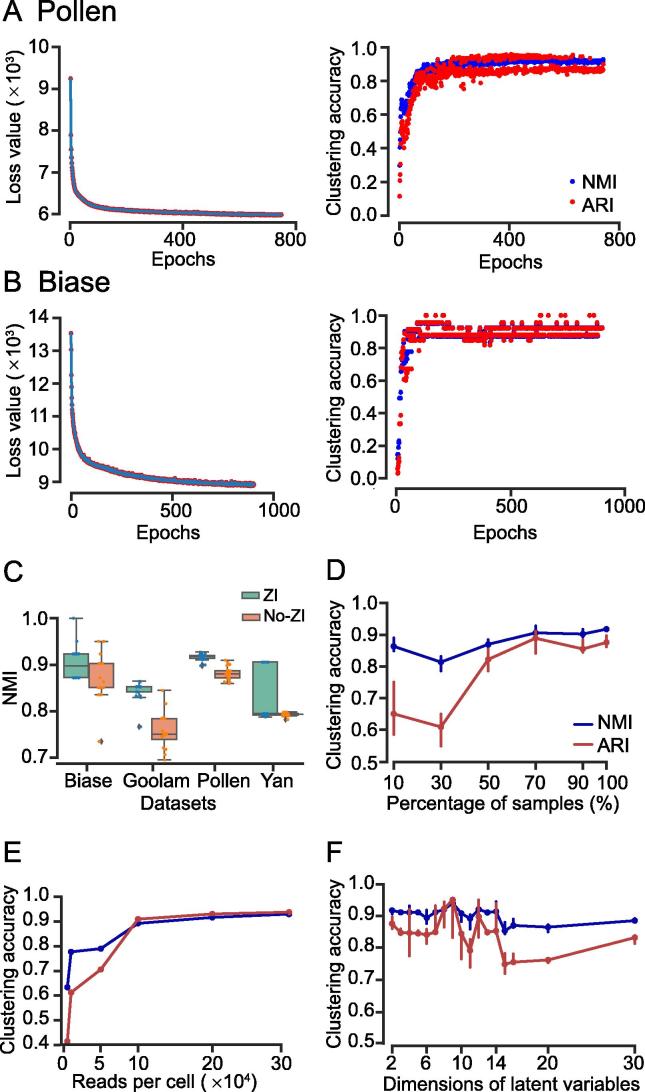
**Analysis of the model stability and parameter settings of VASC** **A.** The iteration process using the Pollen dataset [27]. The change of loss values of the whole network as shown in Equation (2) versus iteration epochs is shown on the left and the right part is the change of NMI and ARI values versus iteration epochs is shown on the right. **B.** The iteration process using the Biase dataset [18]. **C.** The stability of VASC. The boxplots were generated based on 20 repeated runs with (green) or without (orange) the ZI layer. Tests were performed on the Biase [18], Goolam [22], Pollen [27], and Yan [30] datasets. **D.** The down-sampling test on cell numbers based on the Pollen dataset [27]. VASC was run on 10%–100% randomly-sampled cells of the original dataset. **E.** The down-sampling test on read numbers based on the Pollen dataset [27]. **F.** The effects of the dimensions (ranging from 2 to 30) for the latent variables based on the Pollen dataset [27]. ZI, zero-inflated.

In this section, we analyzed the stability and parameter settings of VASC. Firstly, we analyzed the model fitting processes of VASC on two datasets, the Pollen and Biase datasets (with 301 and 56 cells, respectively). Loss function of the whole neural network decreased sharply during the first few epochs, and simultaneously, the NMI and ARI values increased sharply (Figure 4A and B). After the first 100 epochs, the loss curves quickly converged to a lower limit and the loss fluctuations of the dataset with more samples (Pollen) were smaller than those of the dataset with fewer samples (Biase). Based on these observations, VASC is set to stop when there is no obvious decrease in loss function within 50 epochs (see details in the Methods section).

Due to the randomness of the stochastic gradient descent method, the model initialization, and the *k*-means clustering, slightly different results could be generated at different runs. We thus analyzed the four datasets with the smallest sample sizes, including Biase (56 samples), Goolam (124), Pollen (301), and Yan (90), to test the stability of VASC by 20 repeated runs. As expected, the two datasets with relatively more cells (Goolam and Pollen) showed much higher consistent results than the other two datasets (Figure 4C). The NMI values of the Biase dataset were almost distributed between the two boundaries of the boxplots. Considering the relatively small number of cells (only 56 samples), this distribution may be caused by the different clustering output of one or two cells at the boundary between two cell types. A similar result was also observed for the Yan dataset. However, the Goolam and Pollen datasets with more cells did not show this pattern.

Then, the down-sampling experiment based on the Pollen dataset was implemented to further test the effect of number of cells on the stability of VASC. The dataset was bootstrapped with 10%, 30%, 50%, 70%, 90%, and 100% cells, also with 20 repeated runs. Low average NMI and ARI values with high variations were observed when the number of samples was too small. However, comparable NMI and ARI values were achieved when the percentage of sampled cells was above 50% (Figure 4D). We then down-sampled original reads of the Pollen dataset similarly. For each cell, 5000, 10,000, 50,000, 100,000, 200,000, and 300,0000 unique reads were randomly selected for the analysis, following the same pre-preprocessing procedures. As shown in Figure 4E, low NMI and ARI values were observed only when the number of reads was very small.

The ZI layer was incorporated into VASC to model the dropout event. We then evaluated its effectiveness. As shown in Figure 4C, the inclusion of ZI layer improved both the stability and the average performances of VASC on three of the four tested datasets.

The data projection to a 2D subspace is suitable for visualization, but the subspace with higher dimension may explain more variations. To further test the effects of dimension number, we varied the dimensions of the final latent variables from 2 to 20, using the Pollen dataset. Results showed that the increase in the dimensions did not improve the identification of known cell populations and the subspaces with high dimensions may even cause worse performances in terms of NMI and ARI values (Figure 4F).

### Case study: human pre-implantation embryos

**Figure 5:**
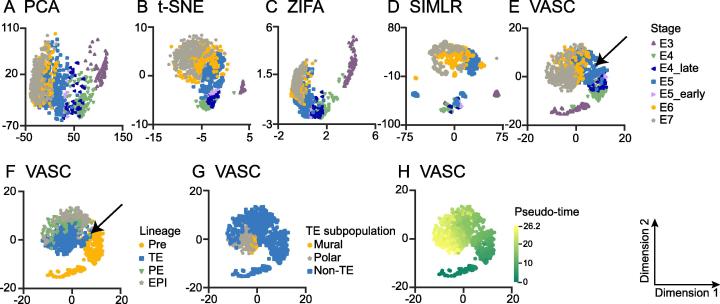
**Visualizations of Petropoulos dataset using different methods and various annotations** The 2D visualization of the Petropoulos dataset using PCA (**A**), t-SNE (**B**), ZIFA (**C**), SIMLR (**D**), and VASC (**E**). Cells are annotated with the developmental stages [18]. **F.** Cells are annotated as pre-lineage and other cells. **G.** TE cells are further annotated as mural and polar cells. **H.** Cells are annotated with the inferred pseudo time. All the annotations are based on the original study [18]. TE, trophectoderm; PE, primitive endoderm; EPI, epiblast.

The scRNA-seq is very useful for studying the cell dynamics during pre-implantation embryo development. We applied VASC on a recently-published dataset of human pre-implantation embryos (the Petropoulus dataset), including 1529 cells with detailed annotations of developmental stages, inferred lineage, and inferred pseudo-time information (all annotations were obtained from original publication) [32]. According to the 2D visualization analysis, VASC and t-SNE recovered the known developmental stages (form E3 to E7) more precisely, with the exception that the E3 cells were out of the trajectory by t-SNE. Both PCA and ZIFA generally recovered the stage trajectory, but the E6 and E7 cells were largely overlapped. SIMLR, which emphasized the modularity of cell populations, did not re-establish the basic pattern (Figure 5A–E).

Compared to t-SNE, a sharper split in the grouping was observed in the E5 cells by VASC (Figure 5B and E). We thus investigated the impact of other annotations on the visualization. We re-annotated the cells with their inferred lineages instead of the developmental stages. Interestingly, we found that the sharp split learned by VASC was a good separation of the pre-lineage cells from the others (Figure 5F). The inner cell mass (ICM), including the primitive endoderm (PE) and epiblast (EPI), were split from trophectoderm (TE), and the boundary was almost perpendicular to the direction of the developmental stage (Figure 5F). Furthermore, the two sub-populations of the TE cells, mural and polar cells, were separated in the visualization as well (Figure 5G). Finally, the trajectory recovered by VASC was strongly coincided with the inferred pseudo time (Figure 5H).

The candidate genes associated with the pre-implantation embryo development were identified by calculating the Spearman’s correlations between the gene expression and the two features shown in the reduced subspace. Many known regulators and markers were found in the top-correlated genes, such as *PGF*, *GCM1*, *CYP19A1*, *MUC15*, *CD24*, *CCR7*, *GREM2*, *CGA*, *GATA2*, *TDGF1*, *ESRG*, *GDF3*, and *DNMT3L* mentioned in the original article [32] (rank ≤100 for either feature). Interestingly, the top-ranked genes were significantly enriched in metabolic processes, such as carbohydrate derivative metabolic process (37 genes, *q* = 5.63E − 05 by DAVID 6.8 [42]), oxidation–reduction process (32 genes, *q* = 4.87E − 05), and lipid metabolic process (32 genes, *q* = 4.94E − 03). Several metabolic pathways have been recently shown to play essential roles in regulating the stemness and differentiation of stem cells [43]. Interestingly, we have identified several candidate genes that are involved in different metabolic processes. These include *CYP11A1* (encoding a member of the cytochrome P450 superfamily of enzymes, the same superfamily of CYP19A1), *NR2F2* (encoding a member of the steroid thyroid hormone superfamily of nuclear receptors), *PKM* (encoding a pyruvate kinase, a key kinase in glycolysis), *PPARG* (encoding a member of the peroxisome proliferator-activated receptor subfamily of nuclear receptors), and *IDH1* (encoding an isocitrate dehydrogenase, a key enzyme for cytoplasmic NADPH production).

### Application on a 10× Genomics dataset

**Figure 6:**
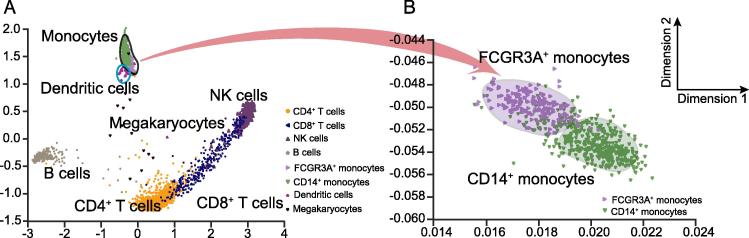
**Application of VASC in the PBMC3k dataset** The 2D visualization of VASC on all cells (**A**) and monocytes (**B**). The PBMC3k dataset was downloaded from 10× Genomics (https://support.10xgenomics.com/single-cell-gene-expression/datasets).

We tested VASC on a dataset called PBMC3k [33] generated using a new scRNA-seq technology – 10× Genomics, which can handle more cells but with a relatively high dropout rate. This dataset contains 2700 cells, each with only ∼2000 unique molecular identifiers (UMIs). The cells were labeled based on computational predictions and known markers. As shown in Figure 6A, VASC can clearly distinguish most cell types, such as B cells, CD4^+^ T cells, CD8^+^ T cells, and NK cells. Cells from common myeloid progenitors, such as dendritic cells, megakaryocytes, and monocytes, were separated from the cells derived from common lymphoid progenitors, like B cells, T cells, and NK cells. Then, we re-ran VASC on the population of monocytes, and consequently further classified them into FCGR3A^+^ monocytes and CD14^+^ monocytes (Figure 6B). Therefore, VASC could identify the major global variance structure in the first place, and then detect subtle differences, when it is restricted to a local cell sub-population. These results indicate that VASC could also perform well for the dataset with more cells and higher dropout rate.

## Discussion

Dimension reduction (or low-dimensional representation) is fundamental to visualization and the downstream analysis of scRNA-seq data. In this study we report VASC, a method based on deep VAE, for dimension reduction and visualization of scRNA-seq data. We evaluate the performance of VASC by comparing with four other commonly-used methods, including PCA, t-SNE, ZIFA, and SIMLR. These methods are broadly divided into two categories. (1) PCA, ZIFA, and VASC aim at finding the representation that can best explain the variations of the original data; and (2) t-SNE and SIMLR try to find another embedded space that can preserve the neighborhood relationship of the samples in the original space. According to our data analysis, the former group of methods can better retain the basic shapes of the data distributions. ZIFA can be treated as a combination of the probabilistic PCA and the zero-inflated model. The major limitation of ZIFA is that it assumes a linear relationship between the hidden subspace and the observed data. Conversely, VASC can deal with complex non-linear patterns based on deep neural networks. Our data show that VASC has better performance than PCA and ZIFA, especially when the sample sizes are larger (Figure 2, Figure 3). The two embedding methods in the latter group, t-SNE and SIMLR, frequently change the topology of the original data space. t-SNE tends to “disperse” the cells in the embedded subspace. Compared to t-SNE, SIMLR adds penalties on the modularity of samples in the embedded subspace, which forces the diagonal-block structure of the learned cell–cell similarity matrix, and tends to generate compact clusters. This penalty is very useful to identify the cell populations with distinct transcriptomes (for examples, the Pollen dataset). Nevertheless, it frequently fails, if the dataset is generated from studies on “continuous” cell developmental processes or cell lineages. Overall, performance evaluation using multiple datasets demonstrates that VASC is superior in most cases and exhibits broader dataset compatibility.

One major application of scRNA-seq is to identify different cell types at a single cell level. According to the quantitative analyses shown in Figure 3, the first two dimensions are enough to capture the major differences between different cells in most cases (NMI >0.7 for 16 out of the 20 datasets tested by VASC). Although higher dimensions can explain more variations in the original datasets, additional variations not associated with cell type (for example, the fluctuations associated with cell cycle) may even reduce the separation of different cell types according to our data analysis. The determination of the optimal dimension is a tricky task if prior knowledge is limited. Usually, higher dimensions should be used when investigating more subtle differences, for example, the intra-cell type heterogeneity.

There are two parameters (the mean vector and the co-variance matrix) in the variational distribution Q(***z***|***X***). When the sample size is small, it is better to fix the co-variance matrix. However, when the size is large enough (>1000 according to our preliminary data analysis), a co-variance matrix learnt from the data can generate better results. It is expected that more complex variational distribution families should be tested in the near future, as the sample size of scRNA-seq dataset is quickly increasing.

We also find that the inclusion of ZI layer improves the representation of VASC in terms of recovering the known cell types. Compared to ZIFA, the Gumbel distribution used by the ZI layer does not generate zeroes strictly, which may additionally model the near-zero dropout events. ZIFA is unable to deal with near-zero events, which could be a limitation of ZIFA [6].

The stochastic optimization algorithms, used in the VASC model learning, introduce variations in the dimension reduction. Repeated runs are thus recommended for more consensus performance, although such random effect is small if the sample size is over several hundreds. The running time is a common issue for deep models. For the large dataset with several thousands of cells, it costs several hours for the VASC model learning using a desktop-level computer with single GPU card, which may be acceptable for most scRNA-seq studies.

## Conclusions

In this study, a dimension reduction method, VASC, was developed for scRNA-seq data visualization and analysis. We systematically compared VASC with four state-of-the-art dimension reduction methods on 20 datasets. Results show that VASC achieves superior performance in most cases and is broadly suitable for different datasets with different data structures in the original space. Especially, VASC could make clearer separation of rare cell types than other methods according to our data analysis. The application on a dataset of the human pre-implantation embryo development shows that VASC can re-establish the cell dynamics in the reduced 2D-subspace and identify the associated marker genes.

## Authors’ contributions

DW and JG designed this study and developed the algorithm. DW made the detailed implementation and performed the data analysis. DW and JG wrote this manuscript. Both authors read and approved the final manuscript.

## Competing interests

The authors declare that they have no competing interests.

## Supplementary material

Supplementary File S1 **Detailed model description and performance assessment of VASC**
