# Supplementary material for: VASC: Dimension Reduction and Visualization of Single-cell RNA-seq Data by Deep Variational Autoencoder
Source: Genomics Proteomics Bioinformatics. 2018 Dec 18;16(5):320–31. doi: 10.1016/j.gpb.2018.08.003 (PMC6364131; doi:10.1016/j.gpb.2018.08.003)
Supplement: Supplementary File S1 — Detailed model description and performance assessment of VASC [file mmc1.pdf]

File S1 Detailed model description and performance assessment of VASC

1 Detailed model description of VASC

Suppose the input expression vector for one cell is denoted as $\boldsymbol{x} \in \mathcal{R}^d$, and suppose this vector has been $\log_2$ transformed (with an addition of 1 to avoid log of zeros) and scaled to $[0, 1]$. The whole network is composed of

- A dropout layer with a high drop-ratio of 0.5
- Encoder network

$$\begin{aligned} \boldsymbol{h}_1 &= \boldsymbol{W_1 x} + \boldsymbol{b_1},\ \boldsymbol{h_1} \in \mathcal{R}^{512} && (1) \\ \boldsymbol{h_2} &= ReLU(\boldsymbol{W_2 h_1} + \boldsymbol{b_2}),\ \boldsymbol{h_2} \in \mathcal{R}^{128} && (2) \\ \boldsymbol{h_3} &= ReLU(\boldsymbol{W_3 h_2} + \boldsymbol{b_3}),\ \boldsymbol{h_3} \in \mathcal{R}^{32} && (3) \end{aligned}$$

  Note we add a L_1 regularization for the first layer's weights $\boldsymbol{W_1}$. $ReLU(x) = \max(0, x)$ is applied elementwise.

- Latent sample

$$\begin{aligned} \boldsymbol{\mu} &= \boldsymbol{W_\mu h_3} + \boldsymbol{b_\mu},\ \boldsymbol{\mu} \in \mathcal{R}^2 && (4) \\ \log \boldsymbol{\Sigma} &= SoftPlus(\boldsymbol{W_\sigma h_3} + \boldsymbol{n_\sigma}),\ \log \boldsymbol{\Sigma} \in \mathcal{R}^2 && (5) \end{aligned}$$

  $Softplus(x) = \log(1 + \exp(x))$ is applied elementwise. We set $\log \boldsymbol{\Sigma} = \boldsymbol{I}$ for datasets with a small number of cells. The sampling procedure is then done by

```python
def sampling(args):
    epsilon_std = 1.0
    z_mean, z_log_var = args

    ## draw samples from standard normal distribution
    epsilon = K.random_normal(shape=K.shape(z_mean),mean=0.,stddev=epsilon_std)

    ## re-parameterization
    return z_mean + K.exp( z_log_var/2 ) * epsilon
```

- Decoder network

$$\boldsymbol{h}_4 = ReLU(\boldsymbol{W_4 z} + \boldsymbol{b_4}),\ \boldsymbol{h_4} \in \mathcal{R}^{32} \tag{6}$$

$$\boldsymbol{h_5} = ReLU(\boldsymbol{W_5 h_4} + \boldsymbol{b_5}),\ \boldsymbol{h_5} \in \mathcal{R}^{128} \tag{7}$$

$$\boldsymbol{h_6} = ReLU(\boldsymbol{W_6 h_5} + \boldsymbol{b_6}),\ \boldsymbol{h_6} \in \mathcal{R}^{512} \tag{8}$$

$$\boldsymbol{h_7} = Sigmoid(\boldsymbol{W_7 h_6} + \boldsymbol{b_7}),\ \boldsymbol{h_7} \in \mathcal{R}^{d} \tag{9}$$

  $Sigmoid(x) = \frac{1}{1+\exp(-x)}$ is applied elementwise.

- Zero-inflated layer: We use $\boldsymbol{p} = \exp(-\boldsymbol{h_7^2})$ as the drop-out probability for each corresponding element. Because the back-propagation algorithm couldn't deal with the binary distribution, we use the Gumbel-softmax distribution and a re-parametration trick to approximate the binary distribution. See details from the papaer. The concrete implementation is as follows

```
def sampling_gumbel(shape,eps=1e-8):
        u = K.random_uniform( shape )
        return -K.log( -K.log(u+eps)+eps )

def compute_softmax(logits,tau):
        z = logits + sampling_gumbel( K.shape(logits) )
        return K.softmax( z / tau )

def gumbel_softmax(args):
        logits,tau = args
        return compute_softmax(logits,tau)
```

  Note for datasets with a large number of cells, we use an annealing strategy for the temperature τ, which is computed as (i is the number of epochs, and τ is updated every 100 epochs)

$$\tau = \min(\tau_0 \exp(-\gamma i), \tau_{min}),\ \gamma = 0.0003 \tag{10}$$

- Loss function: the whole loss function is composed of two parts and is computed as

```
xent_loss = d * metrics.binary_crossentropy(x, x_decoded_mean)
kl_loss = - 0.5 * K.sum(1 + z_log_var - K.square(z_mean) - K.exp(z_log_var), axis=-1)
loss = K.mean(xent_loss + kl_loss)
```

  The first term is the estimated reconstruction error (we used the binary cross-entropy between two distributions defined on [0,1]). Suppose $\boldsymbol{x},\ \boldsymbol{y}$ are the true and predicted values, respectively, and then the binary cross-entropy is computed by

$$-\boldsymbol{x}\log(sigmoid(\boldsymbol{y})) - (1-\boldsymbol{x})\log(1 - sigmoid(\boldsymbol{y})) \tag{11}$$

The second term is the KL divergence between $\mathcal{N}(\boldsymbol{\mu}, \boldsymbol{\Sigma})$ and $\mathcal{N}(\mathbf{0}, \boldsymbol{I})$ with dimension k

$$\mathcal{D}(\mathcal{N}(\boldsymbol{\mu}, \boldsymbol{\Sigma}) || \mathcal{N}(\mathbf{0}, \boldsymbol{I})) = \frac{1}{2}\left(\mathbf{tr}(\boldsymbol{\Sigma}) + \boldsymbol{\mu}^{\boldsymbol{T}}\boldsymbol{\mu} - k - \log\det\boldsymbol{\Sigma}\right) \tag{12}$$

2 Benchmarking

We used built-in fucntions of skelarn package from Python for PCA and t-SNE. Before t-SNE, we first applied PCA to reduce the dimensions to 500 for datasets containing over 500 cells. The key parameter 'perplexity' of t-SNE was set as 0.2 times the number of cells. The code is shown below

```
import numpy as np
from sklearn.decomposition import PCA
from sklearn.manifold import TSNE

## expr is a 2-D array with shape (n_cells,n_features)
pca = PCA(n_components=2).fit_transform(expr)

if expr.shape[1] > 500:
        expr_tsne = PCA(n_components=500).fit_transform(expr)
else:
        expr_tsne = np.copy(expr)
tsne = TSNE(  perplexity=0.2*n_cells  ).fit_transform(expr_tsne)
```

ZIFA [6] package was donwloaded from `https://github.com/epierson9/ZIFA`, and we used their block algorithm

```
from ZIFA import block_ZIFA

## expr is a 2-D array with shape (n_cells,n_features)
Z,_ = block_ZIFA.fitModel(expr,2)
```

SIMLR [7] was installed under the instruction of `https://github.com/BatzoglouLabSU/SIMLR`. We used the following R code to execute it

```
library(SIMLR)

## data is a matrix with shape (n_features,n_cells)
## k is the true number of cell types
y <- SIMLR( data,c=k,cores.ratio = 0 )
```

```
## for larger datasets which cannot be dealed with SIMLR, we used SIMLR_Large_Scale
#y <- SIMLR_Large_Scale( data,c=k )

## Obtain the two-dimension results
ydata <- y[[4]]
```

3 Performance assessment

To measure the quality of low-dimensional representations, k-means clustering was applied to the 2D representations of all the methods, and the clustering results were compared with known cell types provided by their original studies. The number of clusters, k, was set to number of known cell types. Four measure indices were used to assess the performances.

- **Normalized mutual information (NMI)** [37]. Suppose P is the predicted clustering results, and T is the known cell type (the same below). Denote the entropy of P and T as $H(P)$ and $H(T)$, respectively, and the mutual information between them as $\mathrm{MI}(P,T)$. NMI is computed as

$$\mathrm{NMI} = \frac{\mathrm{MI}(P,T)}{\sqrt{H(P)H(T)}} \tag{13}$$

- **Adjusted rand index (ARI)** [38]. Suppose n is the total number of samples, a_i is the number of samples appearing in the i-th cluster of P, b_j is the number of samples appearing in the j-th type of T, and n_{ij} is the number of overlaps between the i-th cluster of P and the j-th type and T. API is computed as

$$\mathrm{API} = \frac{\sum_{ij}\binom{n_{ij}}{2} - \frac{\left[\sum_i \binom{a_i}{2} \sum_j \binom{b_j}{2}\right]}{\binom{n}{2}}}{\frac{1}{2}\left[\sum_i \binom{a_i}{2} + \sum_j \binom{b_j}{2}\right] - \frac{\left[\sum_i \binom{a_i}{2} \sum_j \binom{b_j}{2}\right]}{\binom{n}{2}}} \tag{14}$$

- **Homogeneity(HOM)** [39]. This measure expects that every cluster only contains samples from one cell type. Suppose $H(T|P)$ is the cross-entropy of cell types given the cluster P. The homogeneity score is computed by

$$\mathrm{HOM} = 1 - \frac{H(T|P)}{H(T)} \tag{15}$$

- **Completeness (COM)** [39]. This measure expects that samples from one cell type are assigned to the same cluster, and is computed as

$$\mathrm{COM} = 1 - \frac{H(P|T)}{H(P)} \tag{16}$$

For all the aforementioned measures, larger values (up to 1) mean better performances.

We further used the built-in kmeans function of Python sklearn package for clustering analysis, and used metrics from sklearn to meature the clustering quality.

```
from sklearn.metrics import normalized_mutual_info_score,adjusted_rand_score
from sklearn.metrics import homogeneity_score,completeness_score,silhouette_score
from sklearn.cluster import KMeans
def measure( predicted,true ):
        NMI = normalized_mutual_info_score( true,predicted )
        RAND = adjusted_rand_score( true,predicted )
        HOMO = homogeneity_score( true,predicted )
        COMPLETENESS = completeness_score( true,predicted )
        return {'NMI':NMI,'RAND':RAND,'HOMOGENEITY':HOMO,'COMPLETENESS':COMPLETENESS}

## points is a 2-D array with shape (n_cells,2)
## the initialization function 'kmeans++' may raise exceptions occasionally
kmeans = KMeans( n_clusters=k,n_init=100 ).fit(points)
```

4 Visualization of scRNA-seq datasets

4.1 Baron dataset

This dataset contains a large number of cells from human and mouse pancreas. Totally, there are 4 human donors with 1937, 1724, 3605, and 1303 cells, and 2 mice with 822 and 1064 cells, respectively [17].

4.1.1 Baron-human-1

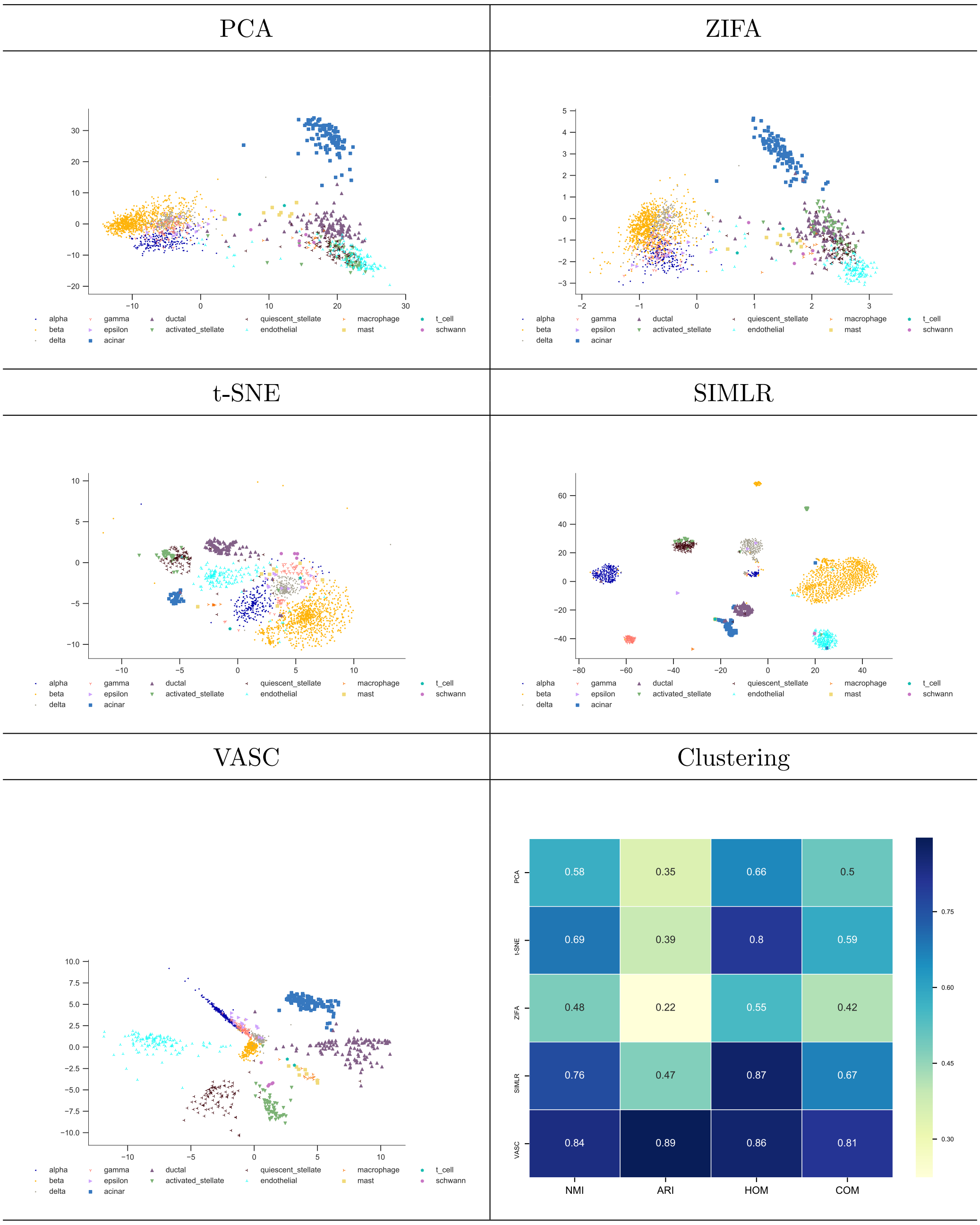

4.1.2 Baron-human-2

PCA

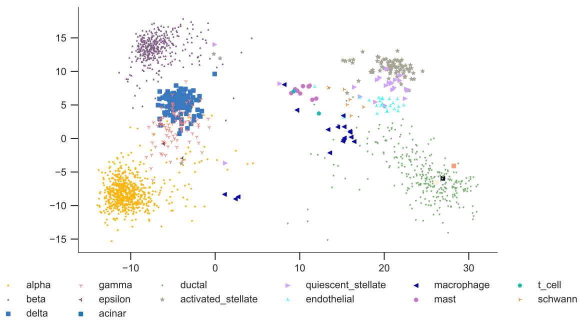

ZIFA

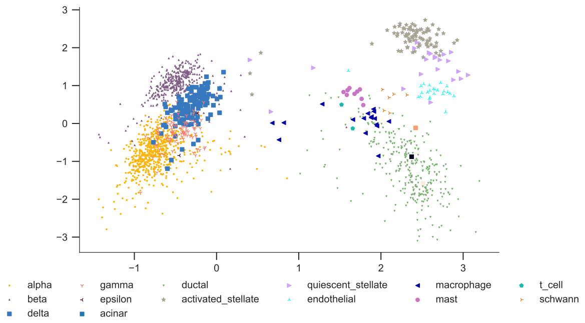

t-SNE

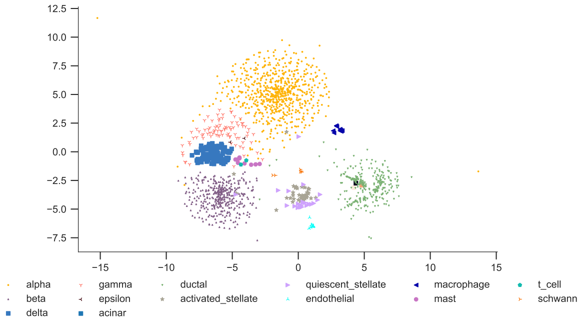

SIMLR

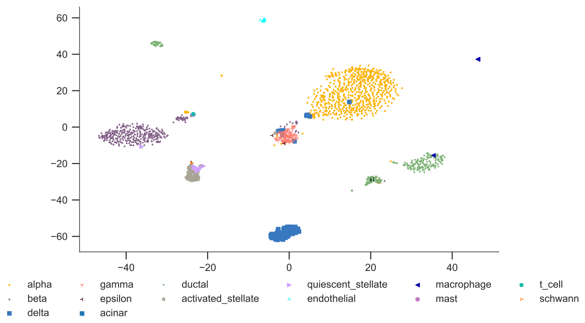

VASC

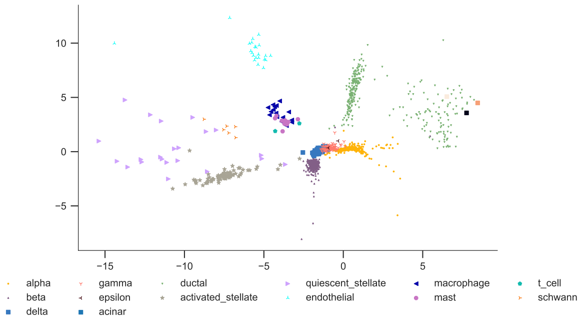

Clustering

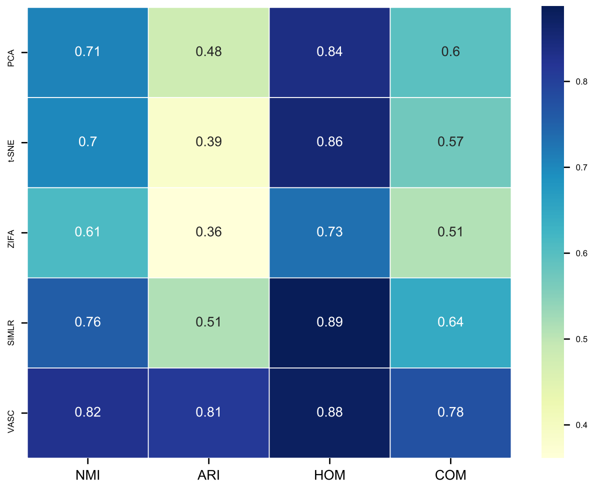

4.1.3 Baron-human-3

PCA

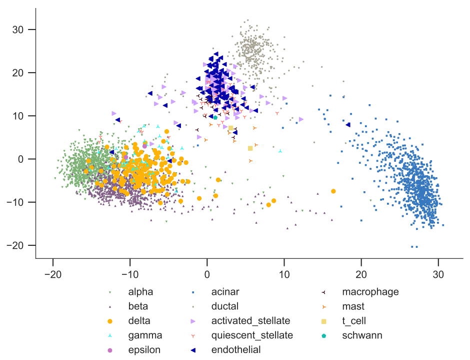

ZIFA

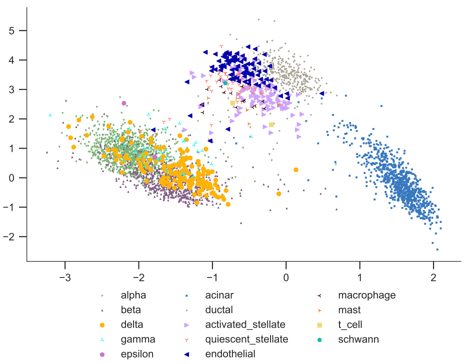

t-SNE

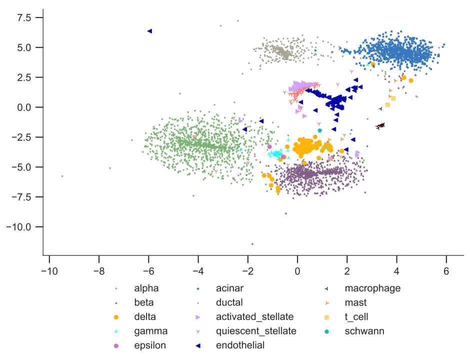

SIMLR

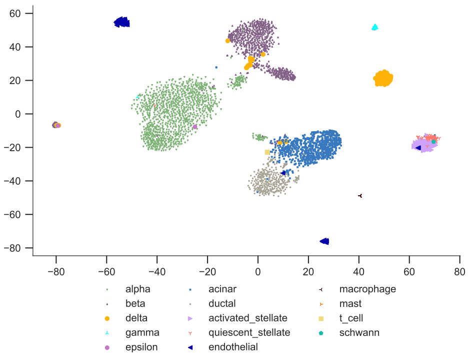

VASC

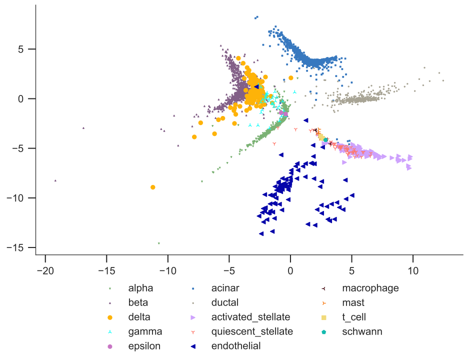

Clustering

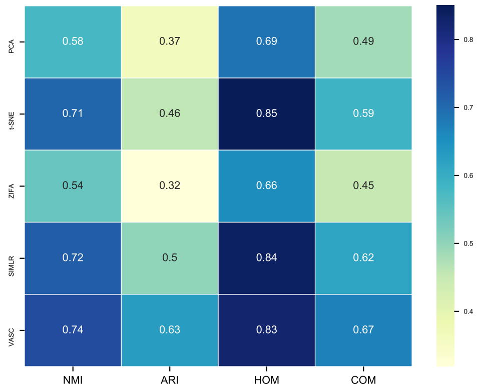

4.1.4 Baron-human-4

PCA

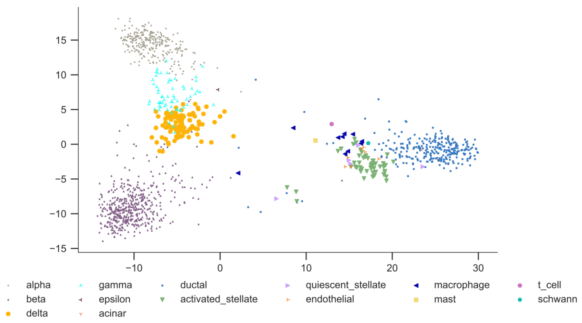

ZIFA

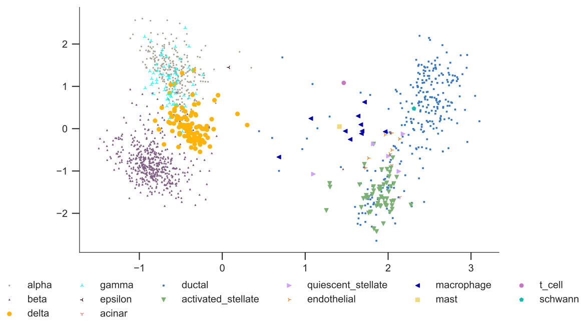

t-SNE

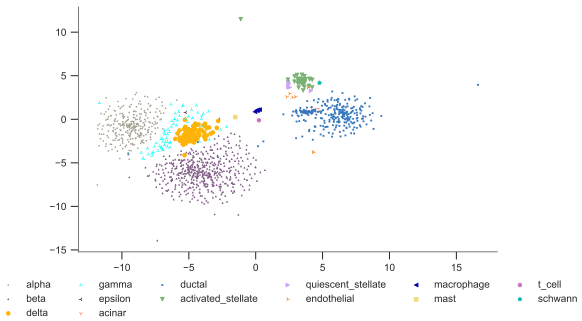

SIMLR

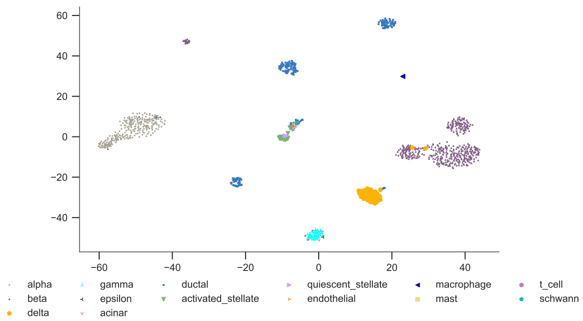

VASC

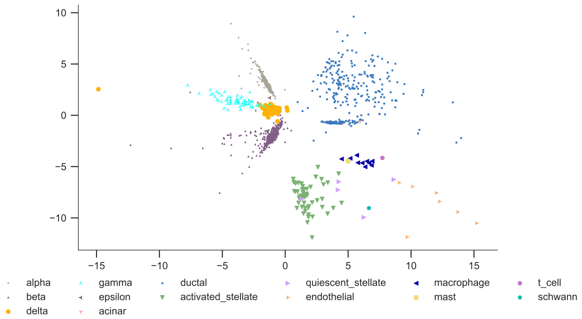

Clustering

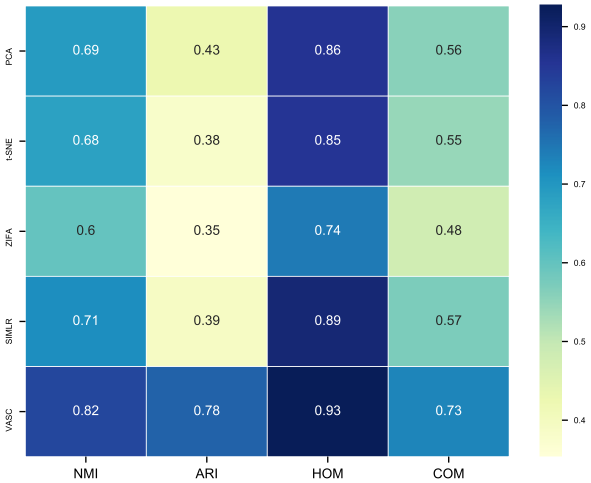

4.1.5 Baron-mouse-1

PCA

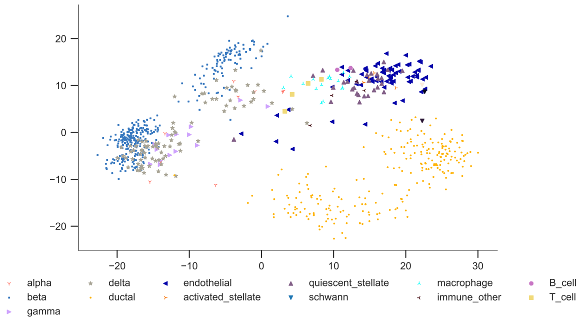

ZIFA

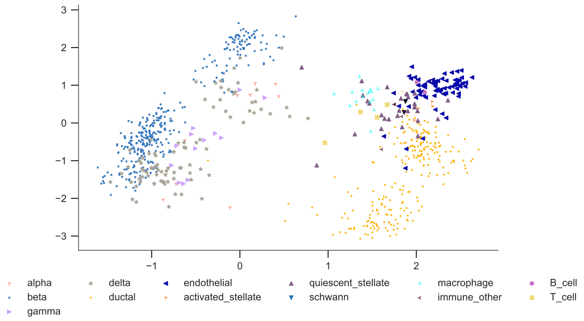

t-SNE

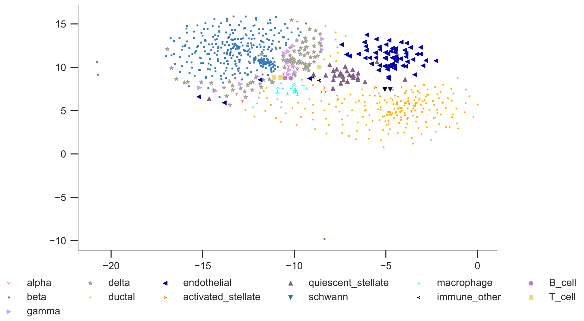

SIMLR

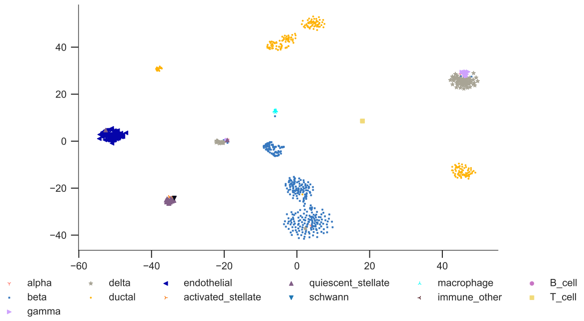

VASC

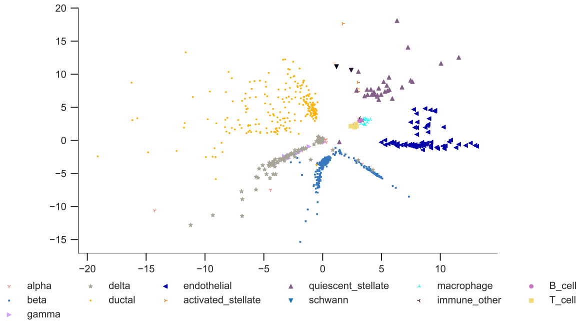

Clustering

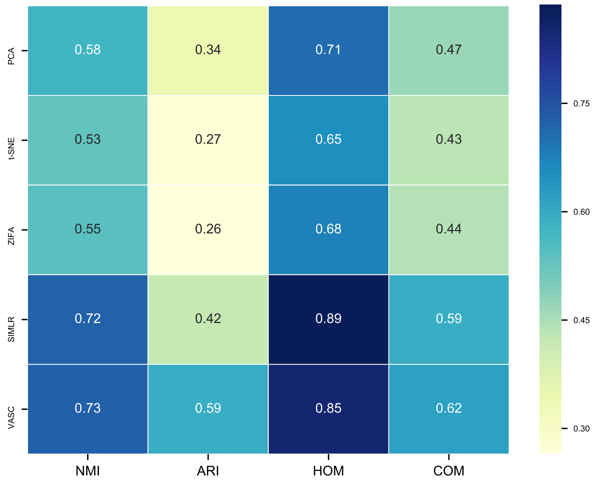

4.1.6 Baron-mouse-2

PCA

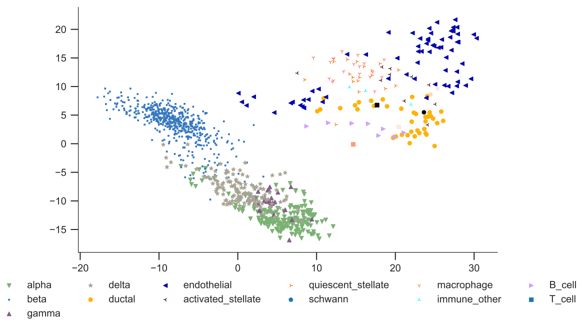

ZIFA

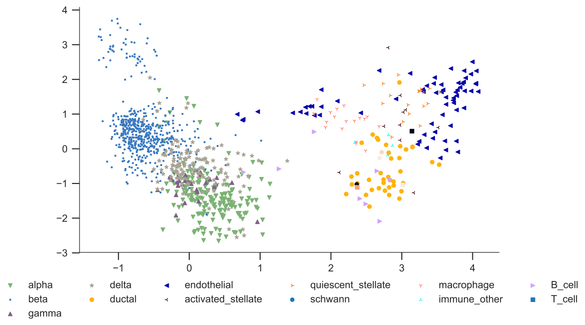

t-SNE

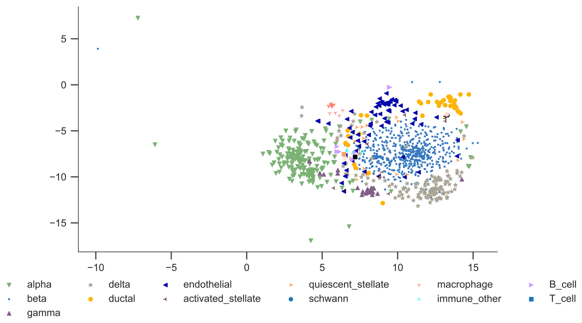

SIMLR

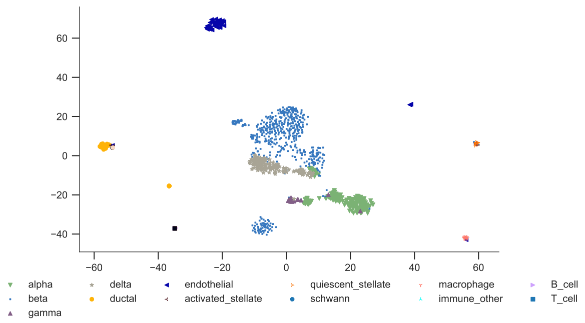

VASC

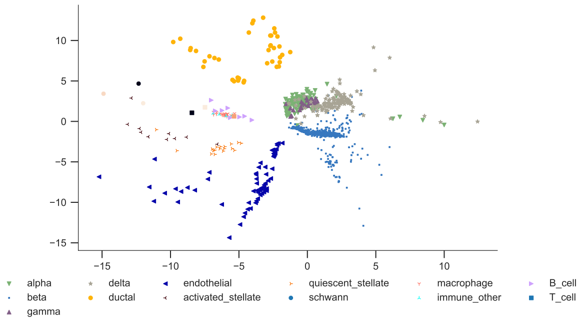

Clustering

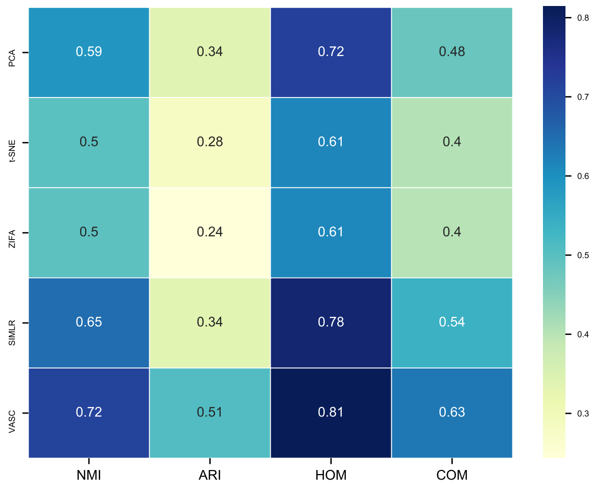

4.2 Biase dataset

This dataset contains mouse embryonic cells, covering cell stages including zygote, 2-cell, 4-cell, and blast, with 56 cells in total [18].

PCA

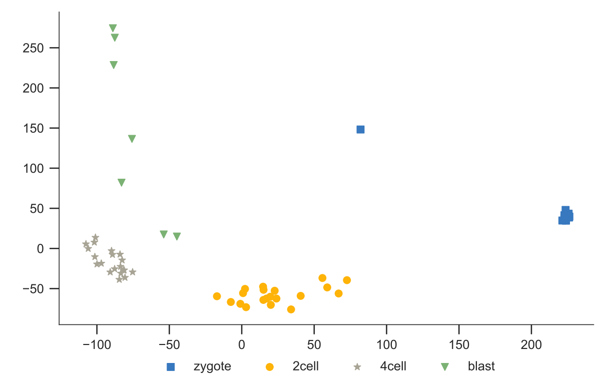

ZIFA

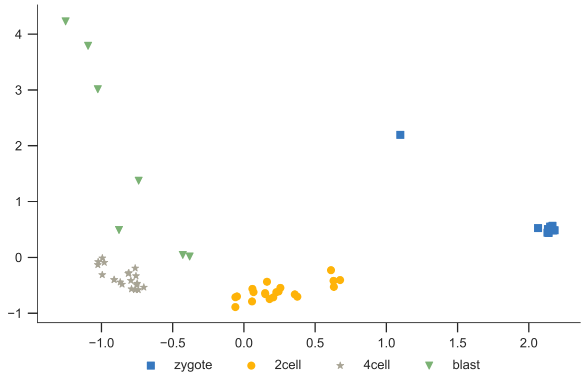

t-SNE

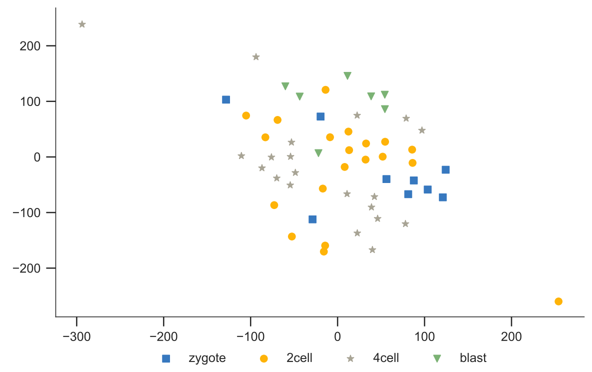

SIMLR

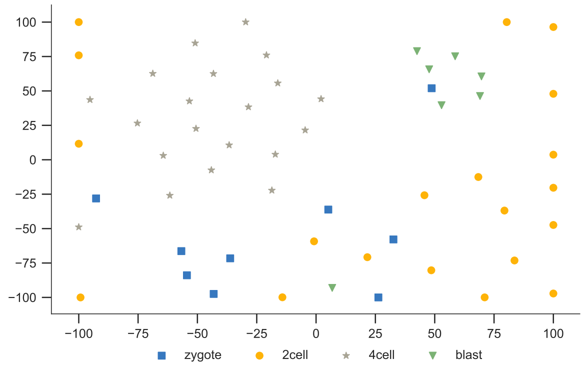

VASC

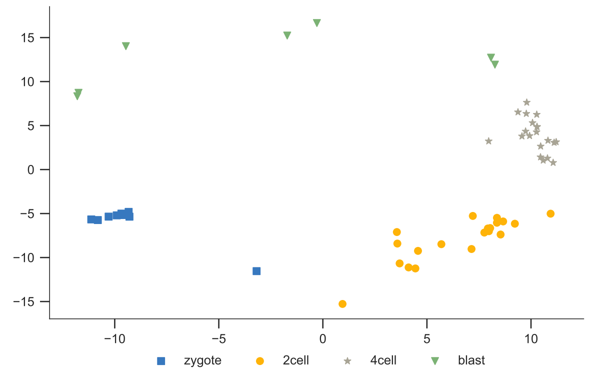

Clustering

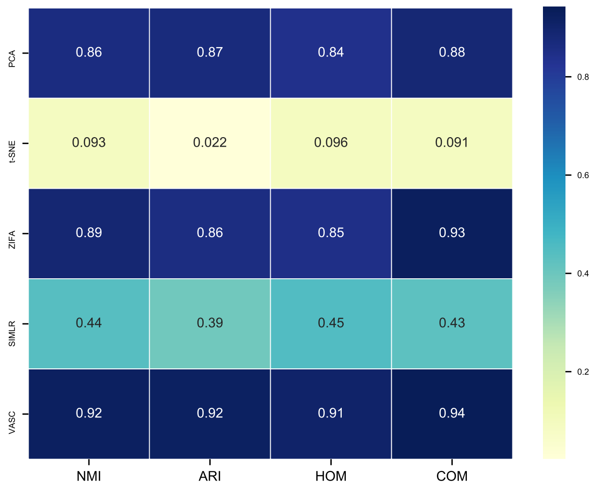

4.3 Camp dataset

This dataset contains 777 cells about human liver development from pluripotency [19]. These cells were sampled at different time points during hepatic cell differentiation: iPS cells, definitive endoderm, hepatic enoderm, immature hepatoblast, and mature hepatocyte. VASC arranged the cells in the same order. Another two cell types - endothelial and mesenchymal are supportive cells. VASC and SIMLR clustered these cells into two sub-populations seperately.

PCA	ZIFA
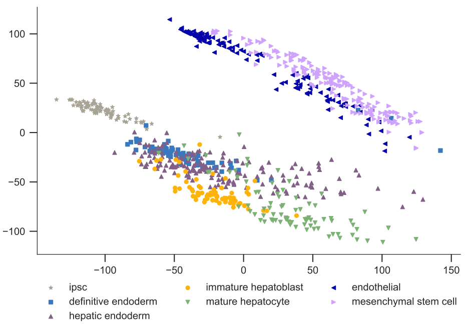	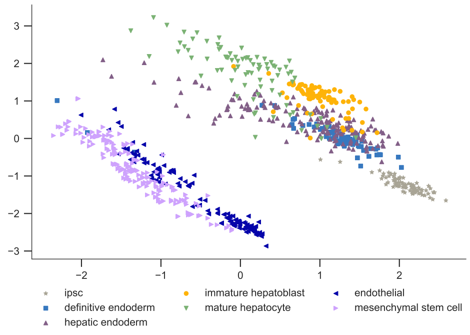
**t-SNE**	**SIMLR**
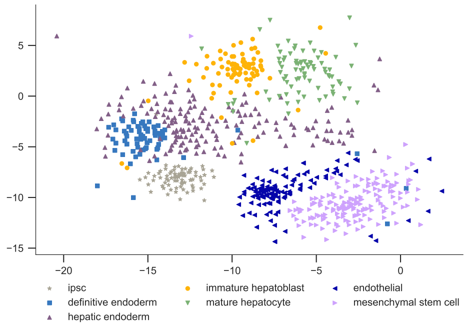	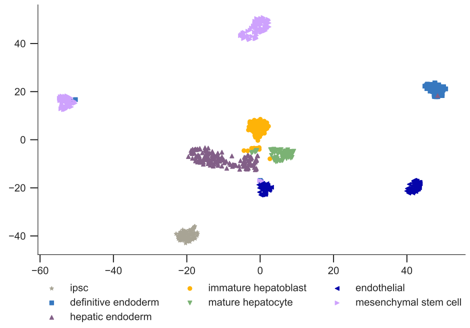
**VASC**	**Clustering**
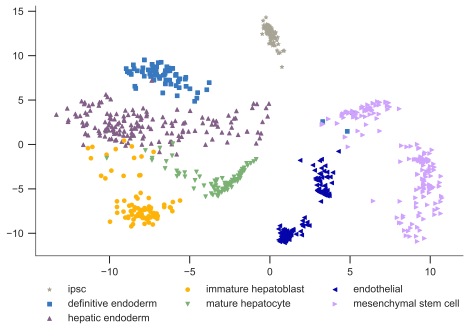	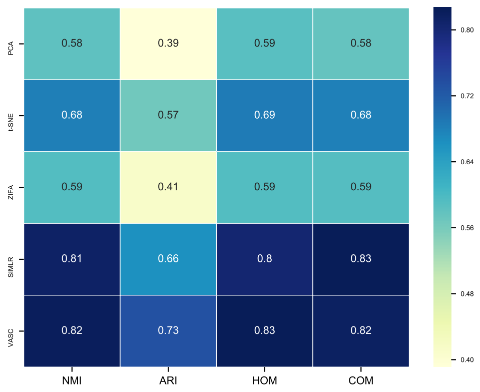

4.4 Darmanis dataset

This dataset contains 466 cells from human cerebral cortex [20].

PCA

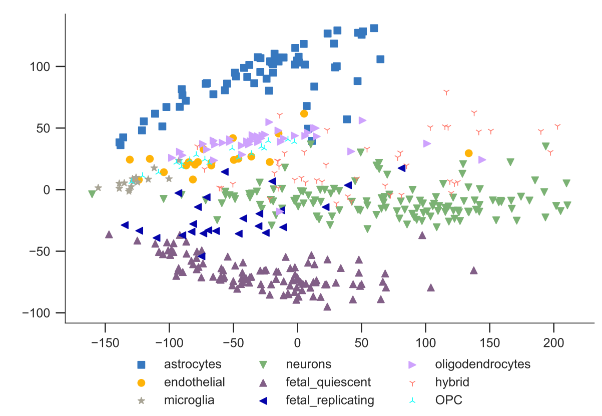

ZIFA

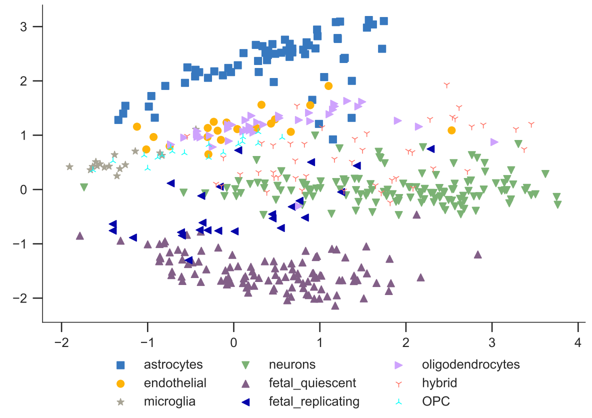

t-SNE

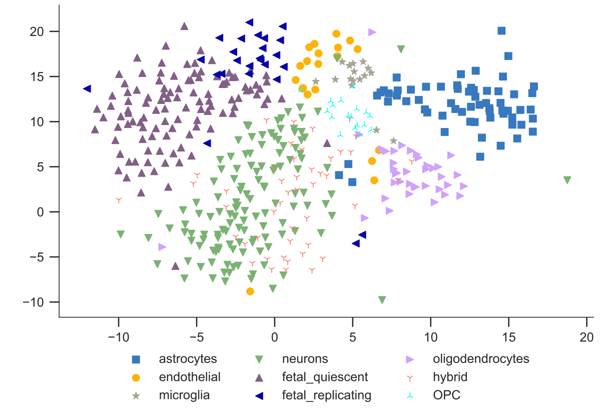

SIMLR

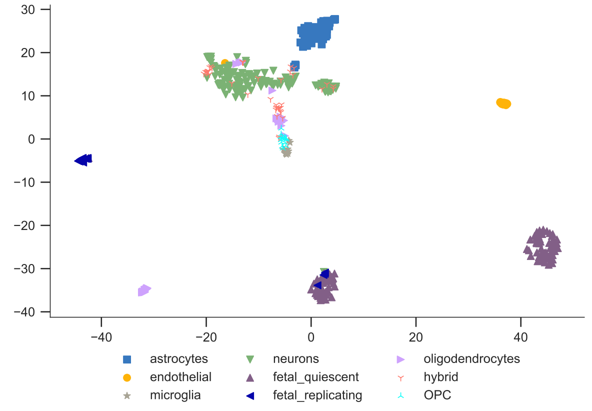

VASC

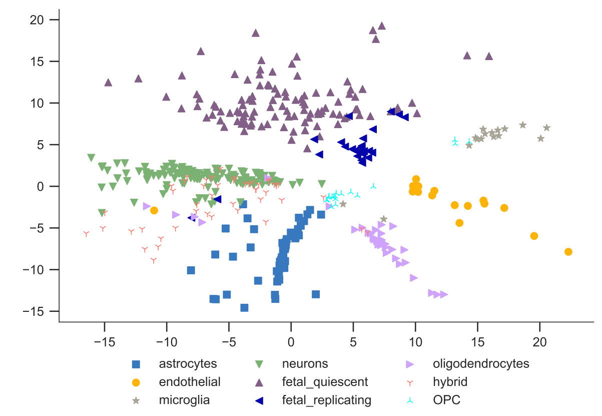

Clustering

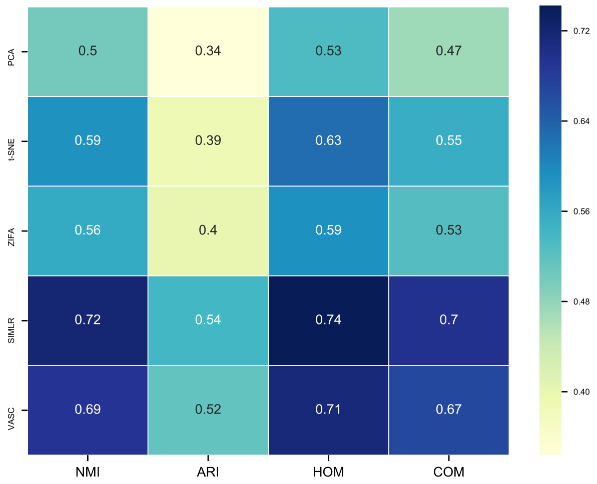

4.5 Deng dataset

This dataset also contains mouse embryonic cells, with 268 cells in total covering six stages including zygote, 2-cell, 4-cell, 8-cell, 16-cell, and blast [21].

PCA

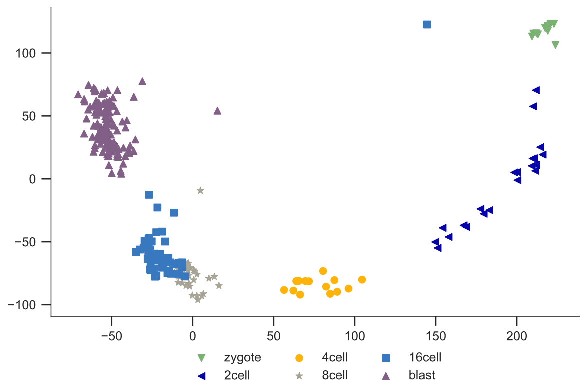

ZIFA

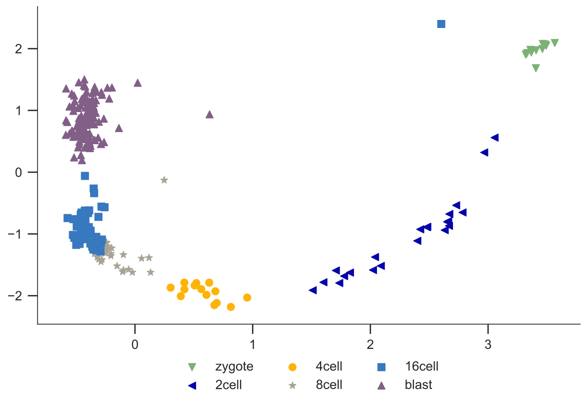

t-SNE

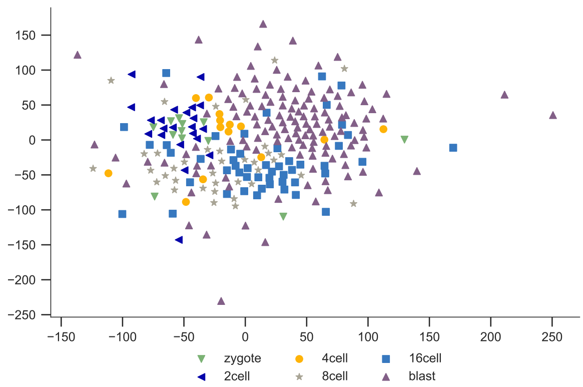

SIMLR

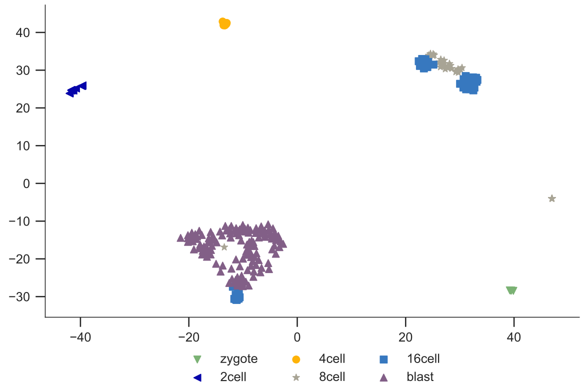

VASC

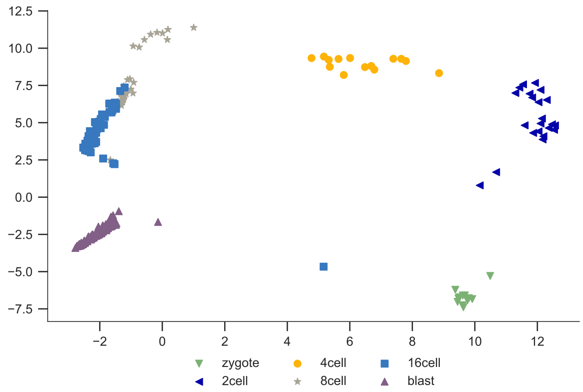

Clustering

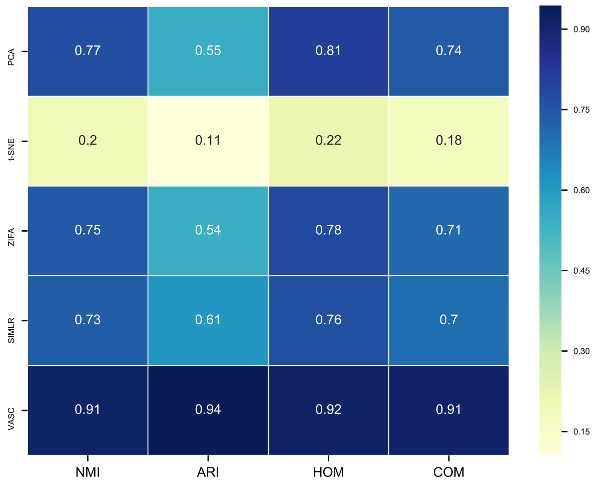

4.6 Goolam dataset

This dataset also contains mouse embryonic cells, covering cell stages including 2-cell, 4-cell, 16-cell, and blast, with 124 cells in total [22].

PCA	ZIFA
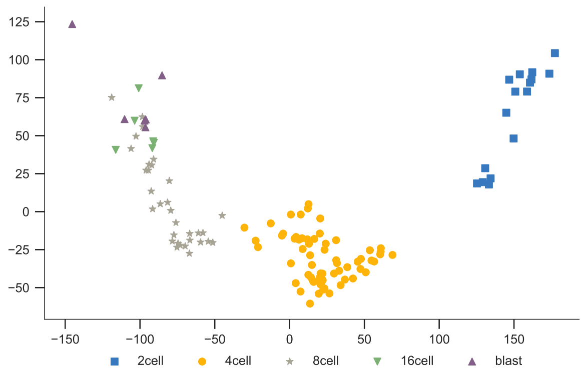	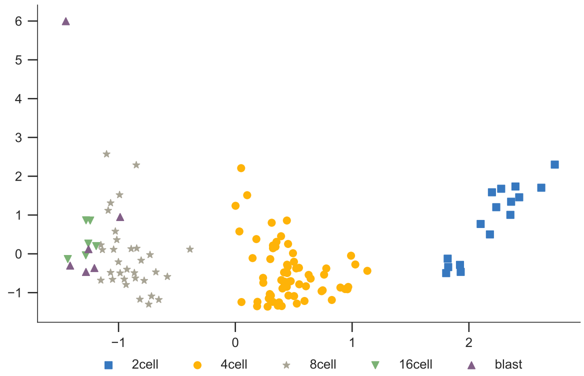
**t-SNE**	**SIMLR**
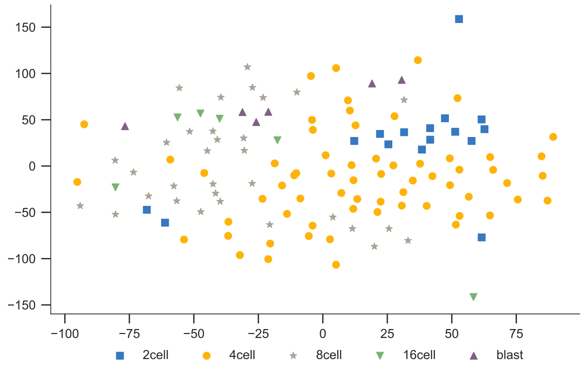	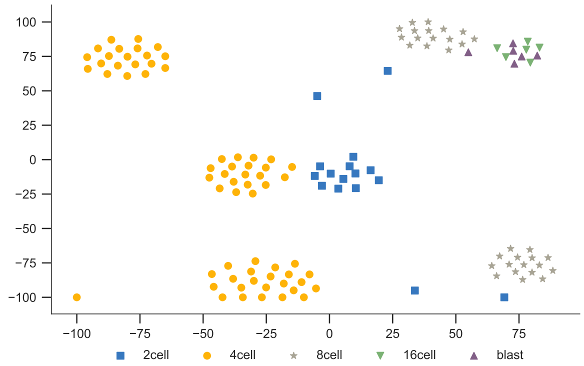
**VASC**	**Clustering**
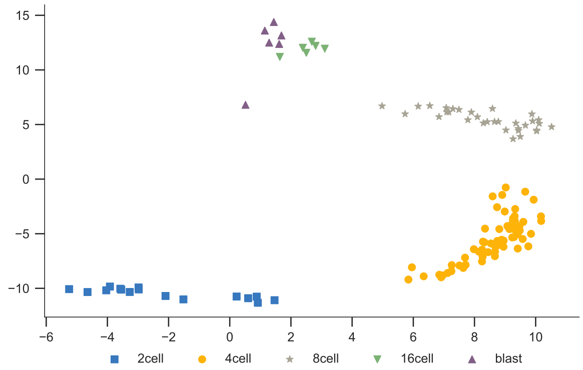	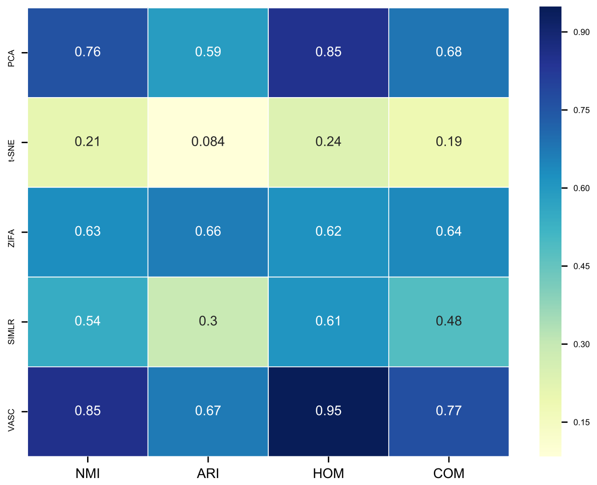

4.7 Klein dataset

This dataset contains 2717 mouse embryonic stem cells, which were sequenced by droplet barocoding [23]. VASC and PCA split d7 into two parts.

PCA

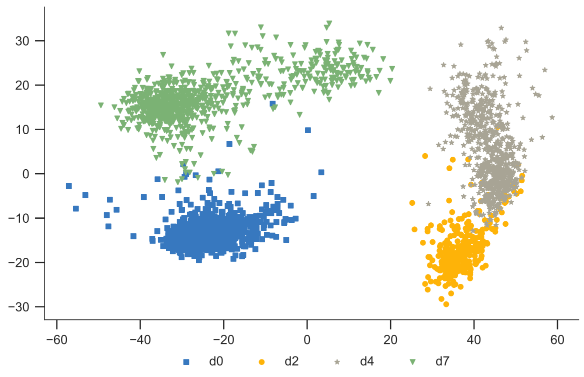

ZIFA

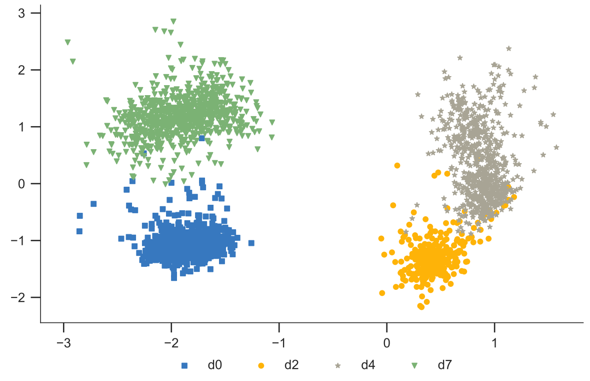

t-SNE

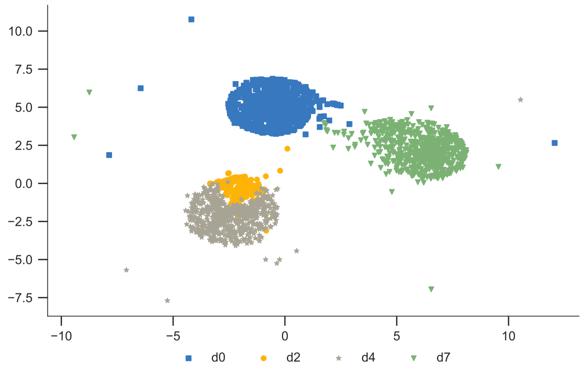

SIMLR

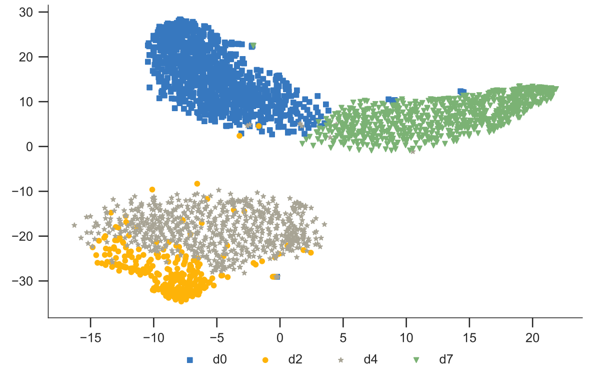

VASC

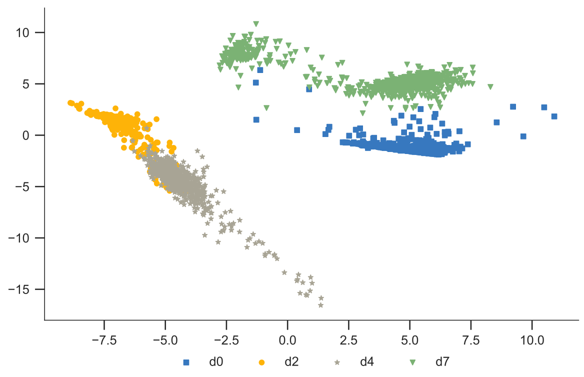

Clustering

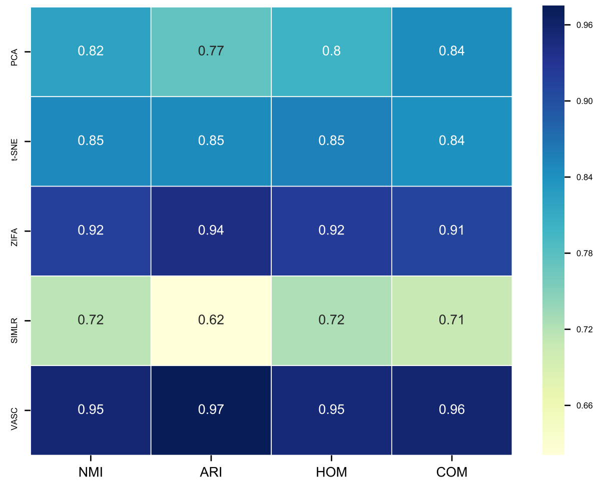

4.8 Kolodziejczyk dataset

This dataset contains cells from three different embryonic stem cell culture conditions: lif(serum), 2i, and alternative ground state 2i. Every cell type also contains data from different chips [24].

PCA

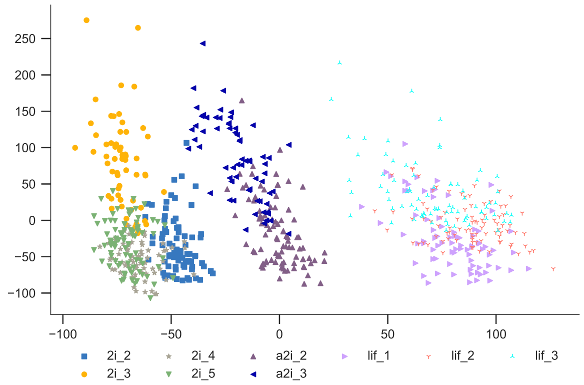

ZIFA

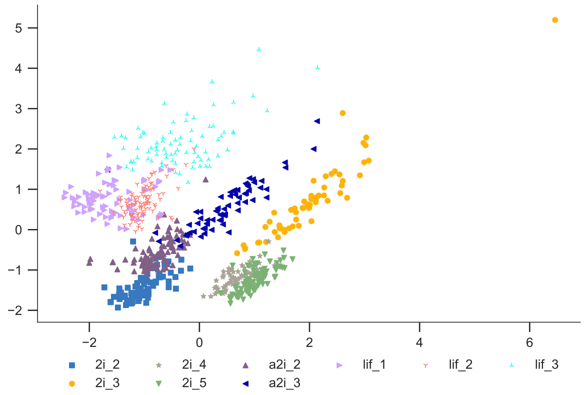

t-SNE

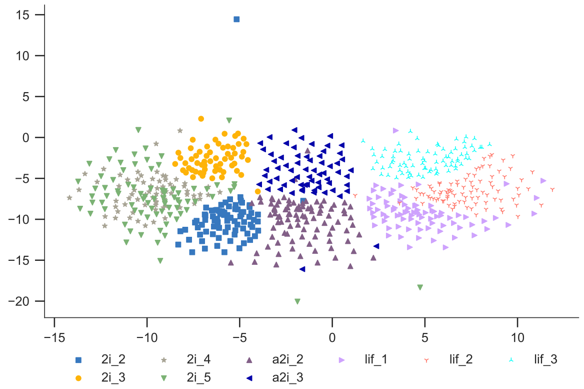

SIMLR

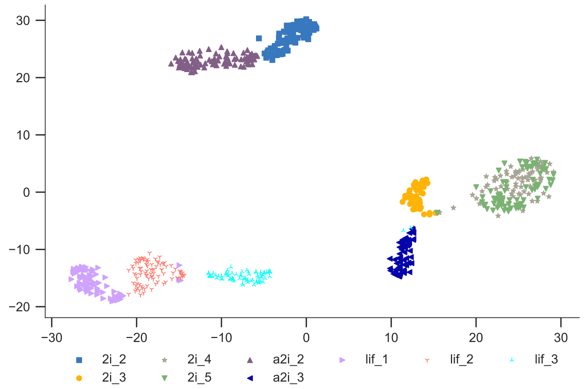

VASC

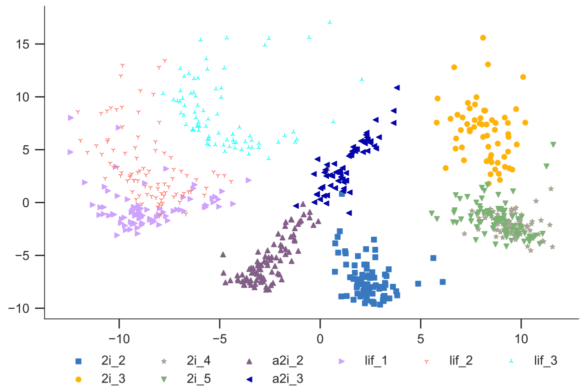

Clustering

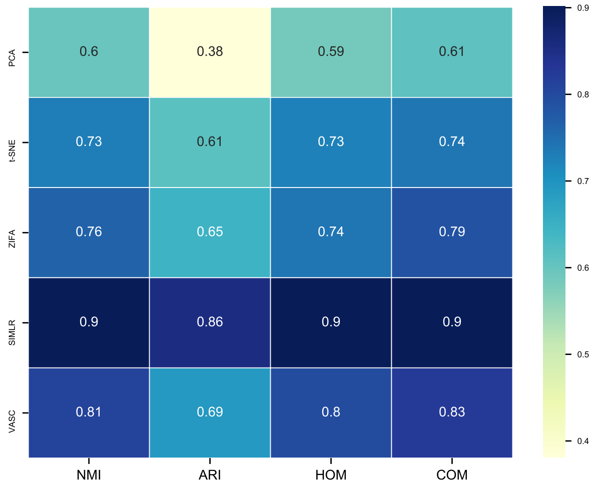

4.9 Li dataset

This dataset contains 561 cells from human colorectal tumors [25]. We found SIMLR achieved almost perfect spliting of these cells, while comparable results were also obtained using VASC.

PCA

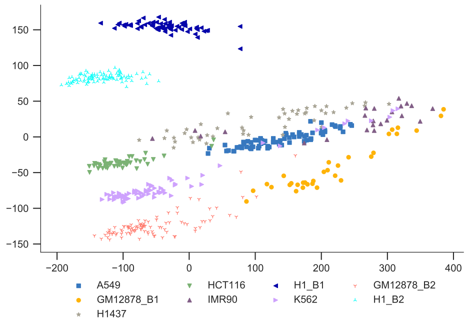

ZIFA

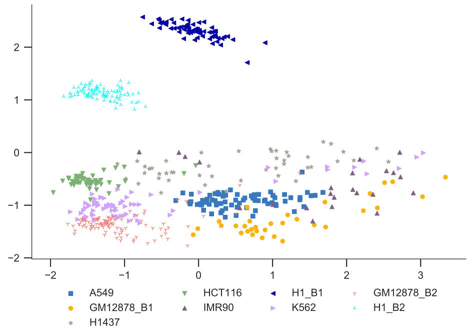

t-SNE

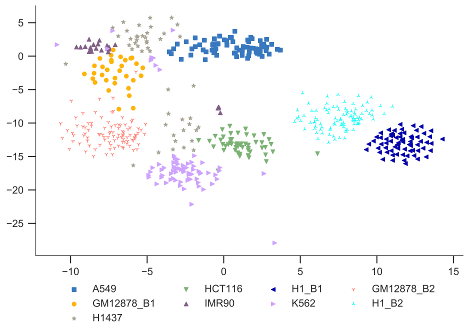

SIMLR

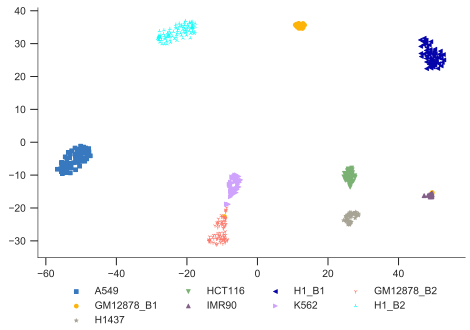

VASC

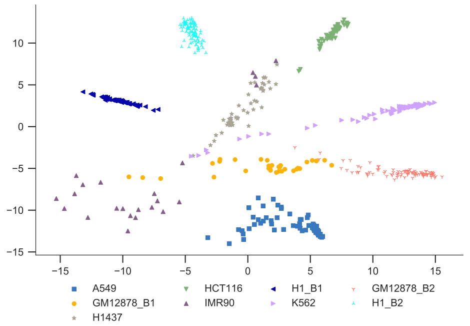

Clustering

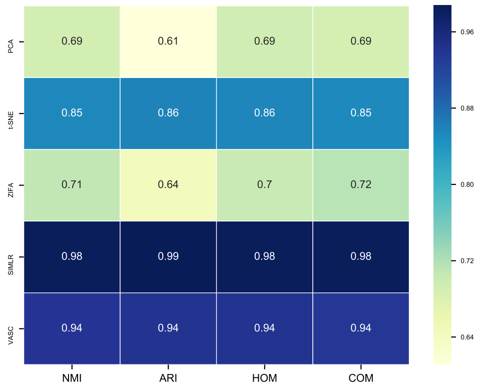

4.10 Patel dataset

This dataset contains 430 cells from five primary glioblastoma [26]. Only VASC and SIMLR could split these cells apart.

PCA	ZIFA
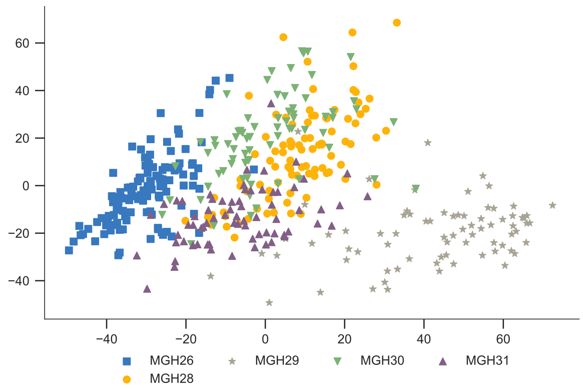	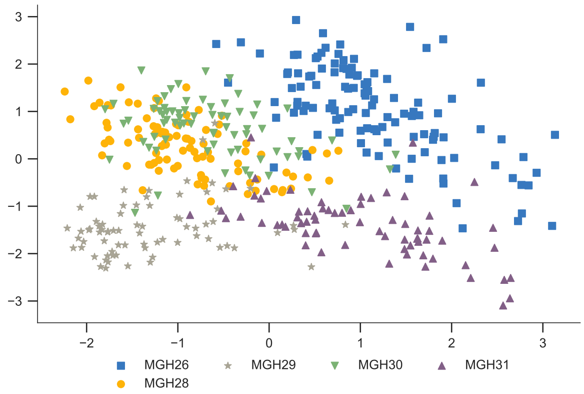

t-SNE	SIMLR
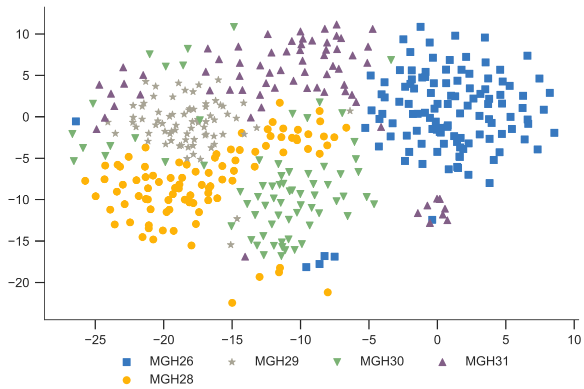	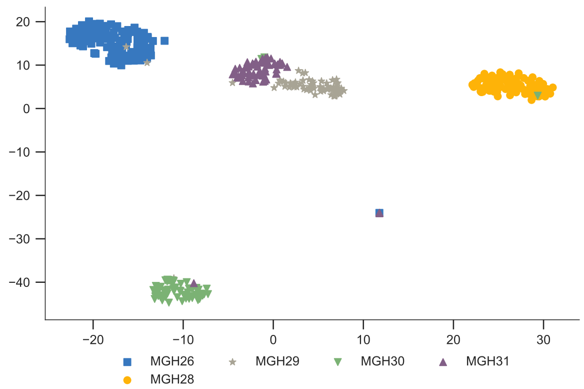

VASC	Clustering
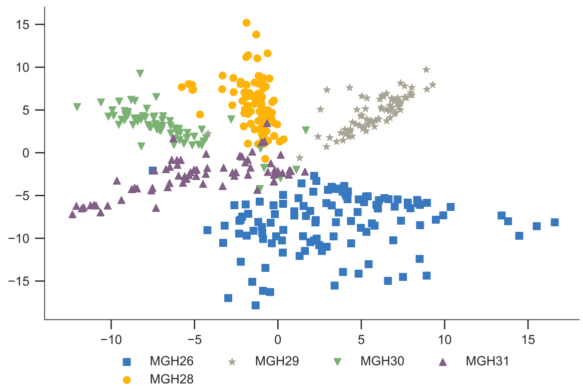	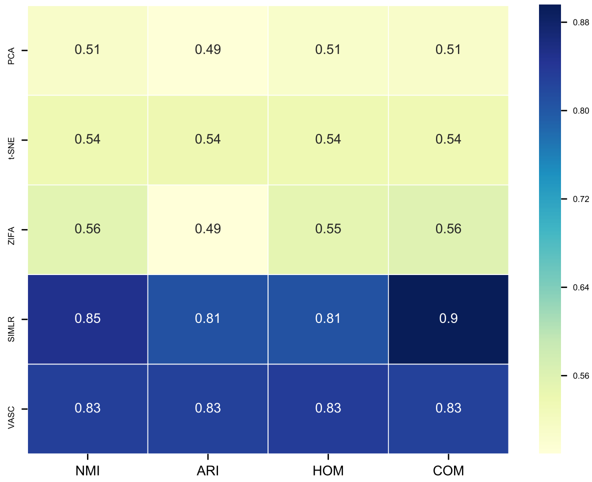

4.11 Petropoulos dataset

This dataset contains human embryonic cells at different development time points, and lineage stages. There are 1529 cells in total [32]. This dataset is hard to cluster because cells may vary in a continuous space.

PCA	ZIFA
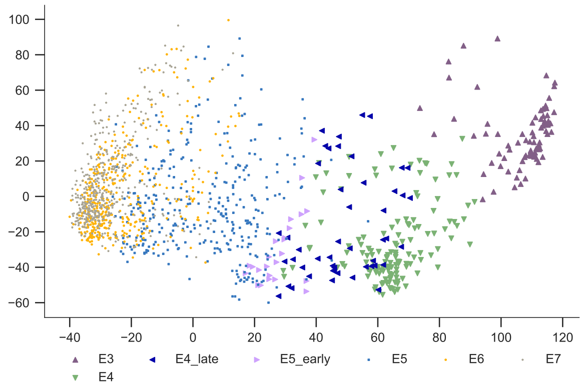	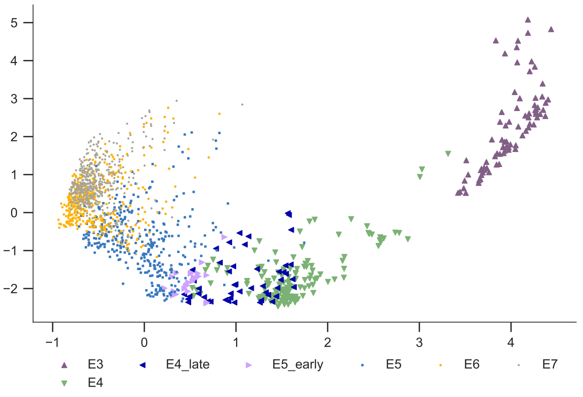
**t-SNE**	**SIMLR**
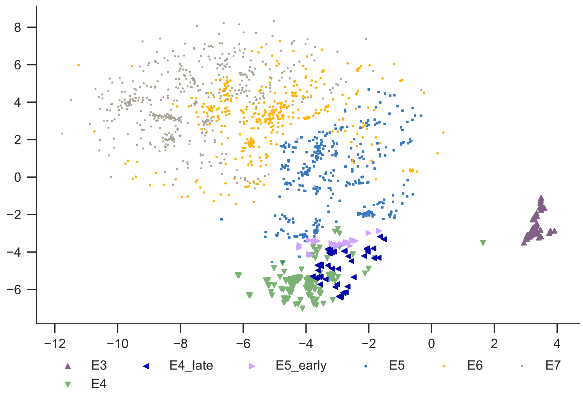	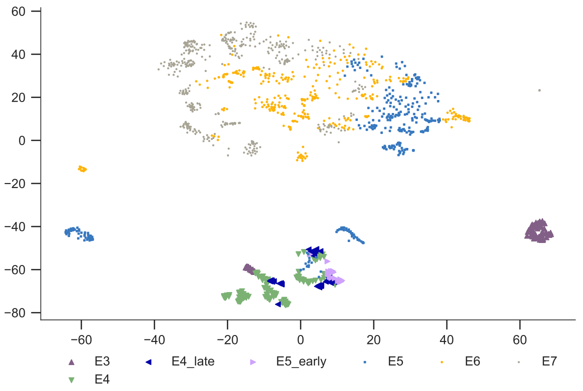
**VASC**	**Clustering**
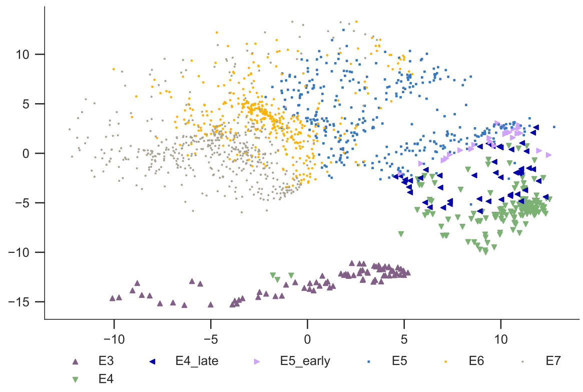	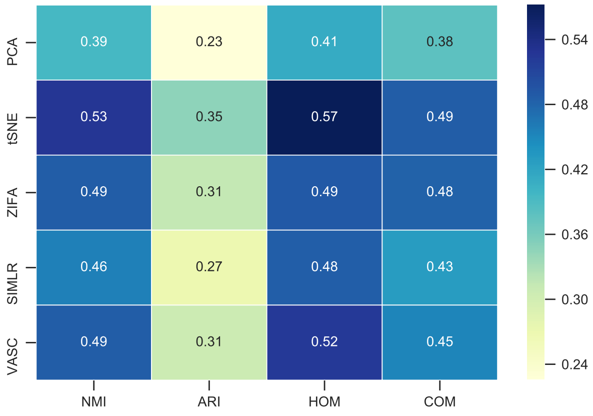

4.12 Pollen dataset

This dataset contains diverse cell types, including skin cells, blood cells, pluripotent stem cells, and neural cells. There are 301 cells in total, from 11 different cell types [27].

PCA

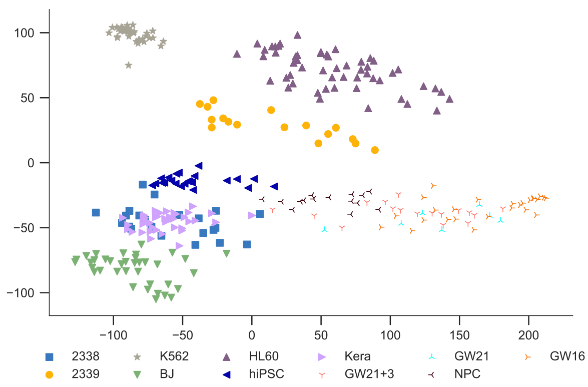

ZIFA

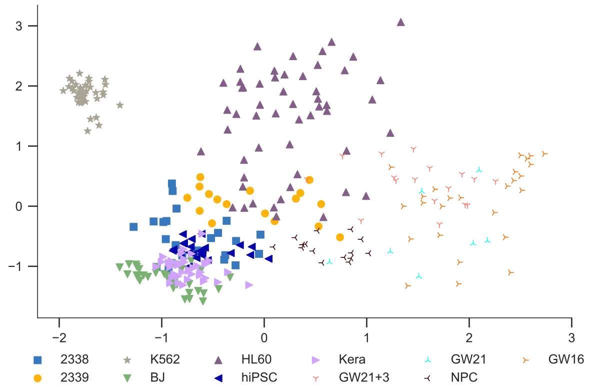

t-SNE

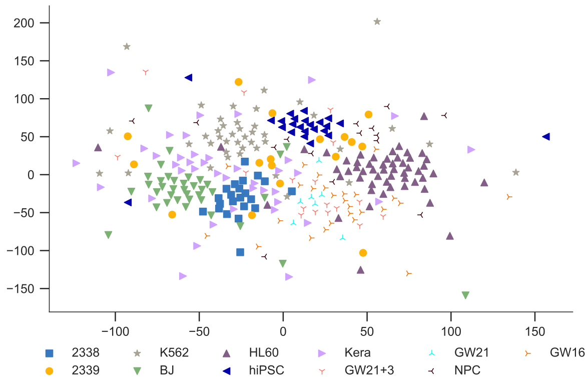

SIMLR

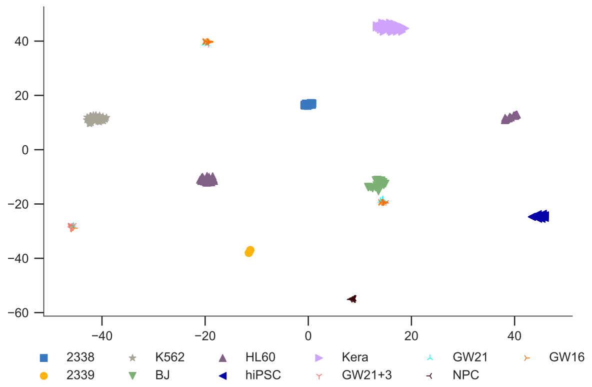

VASC

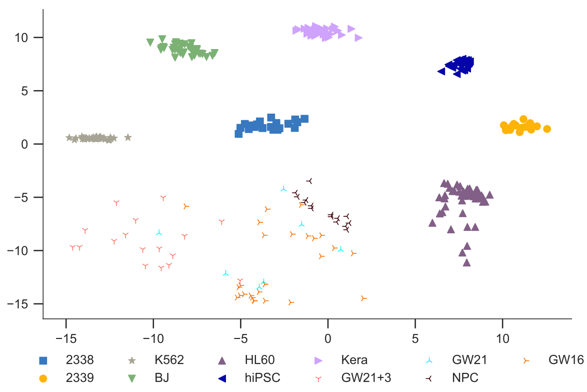

Clustering

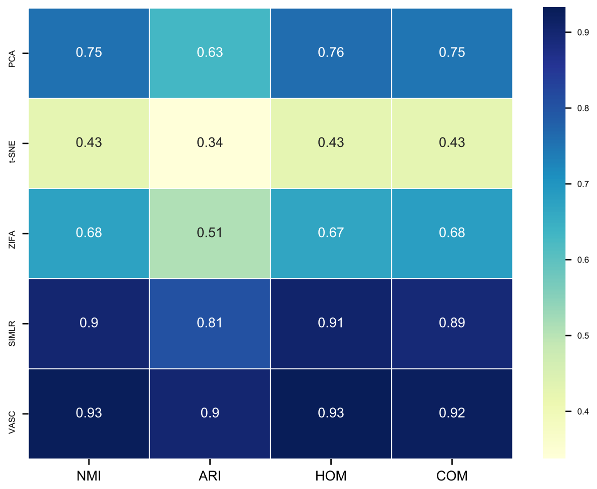

4.13 Usoskin dataset

This dataset contains 622 cells from different sensory neuron types [28]. Usoskin et al. used computational methods to perform cell classification. There are 11 types of cells: 5 NF clusters (expressing neurofilament heavy chain), 3 NP clusters (non-peptidergic nociceptors), 2 PEP clusters (peptidergic nociceptors), and a TH cluster (tyrosine hydroxylase containing). None of our tested methods could distinct these 11 clusters. The original study found no heterogeneity in the TH cluster. VASC and SIMLR could seperate TH from others, while SIMLR made two clusters of TH.

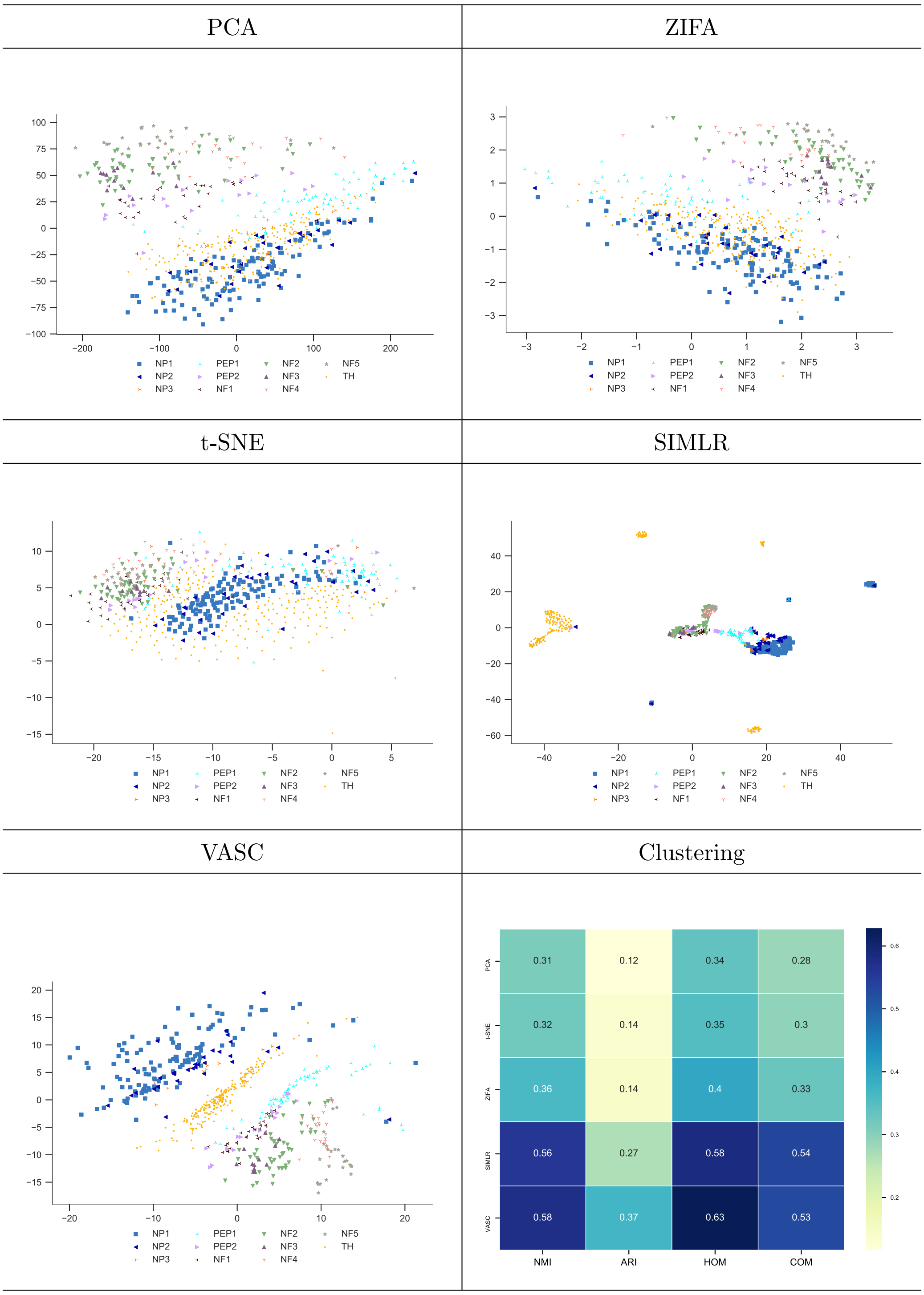

4.14 Xin dataset

This dataset contains 1600 cells from human pancreatic islets, which were sampled from 12 non-diabetic donors and 6 type II diabetic donors [29].

PCA

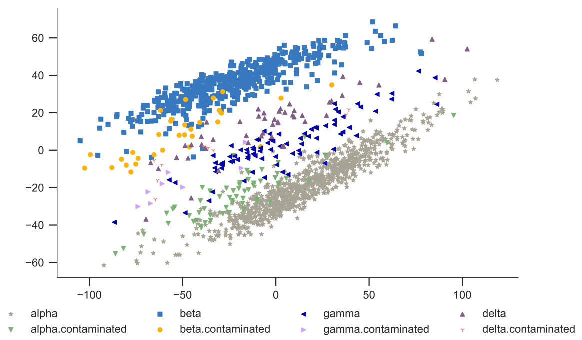

ZIFA

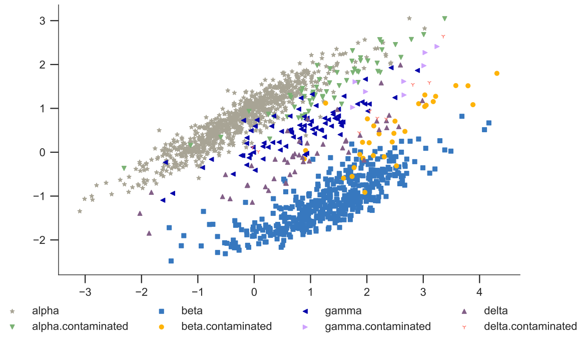

t-SNE

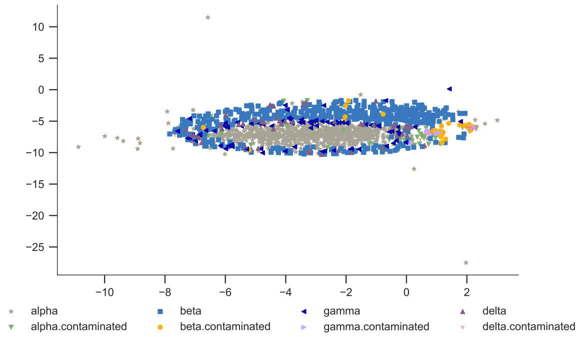

SIMLR

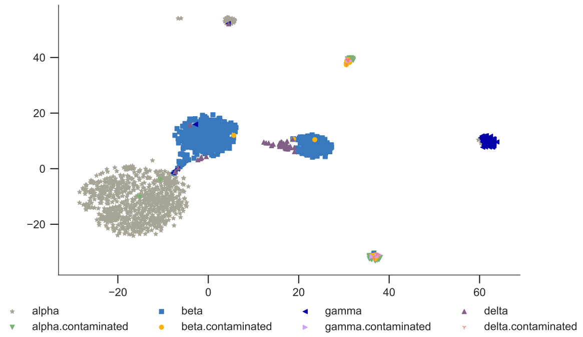

VASC

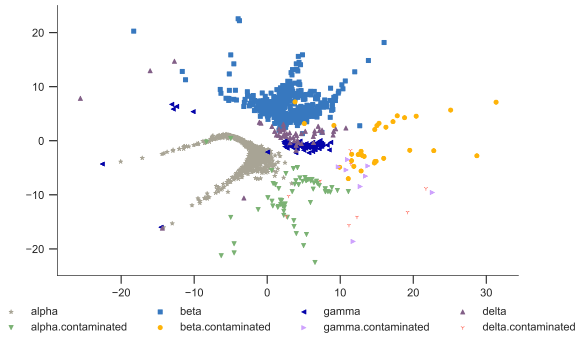

Clustering

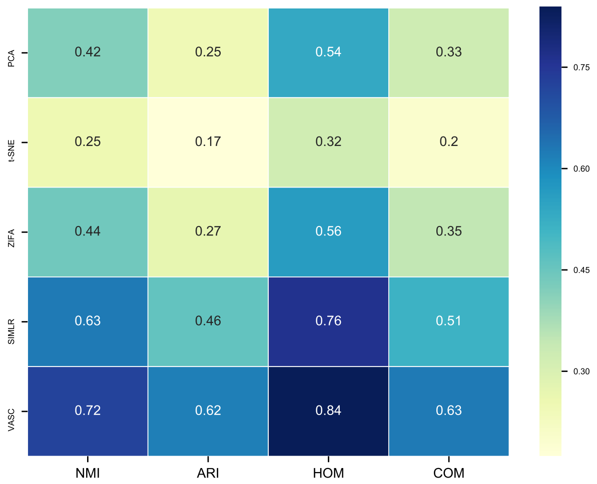

4.15 Yan dataset

This is also a dataset containing mouse embryonic cells with stages including zygote, 2-cell, 4-cell, 8-cell, 16-cell, and blast. There are 90 cells in total [30].

PCA

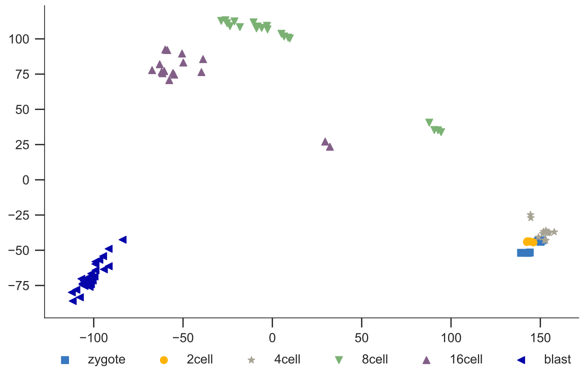

ZIFA

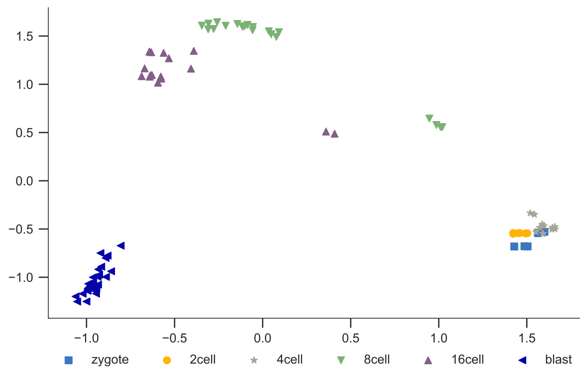

t-SNE

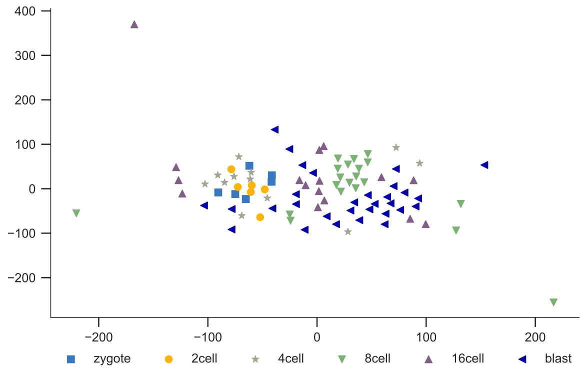

SIMLR

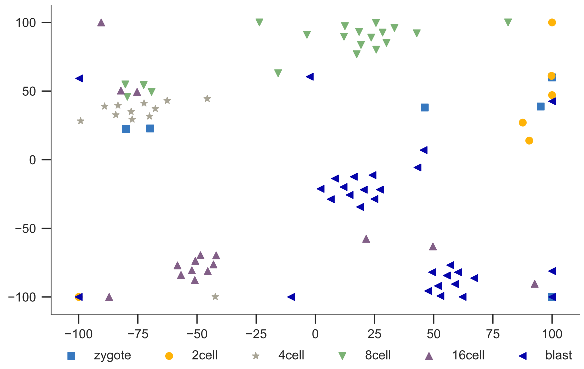

VASC

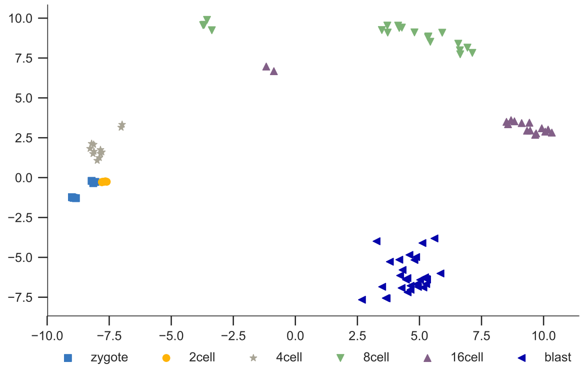

Clustering

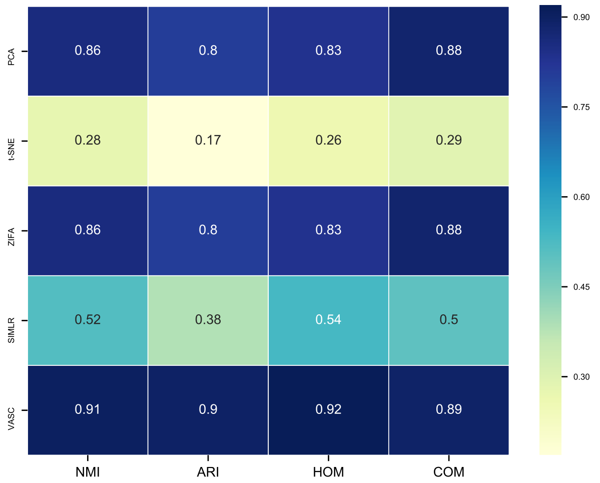

4.16 Zeisel dataset

This dataset contains 3005 cells from 9 cell types of mouse brain [31].

PCA	ZIFA
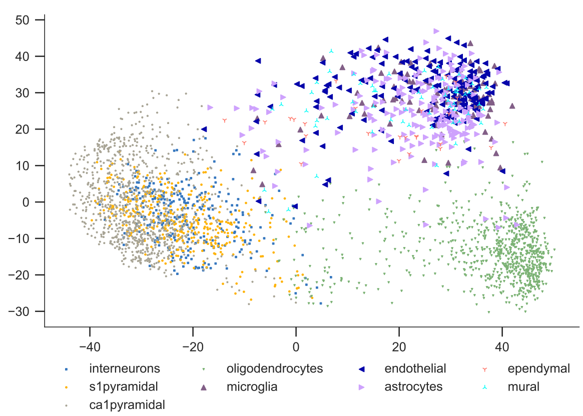	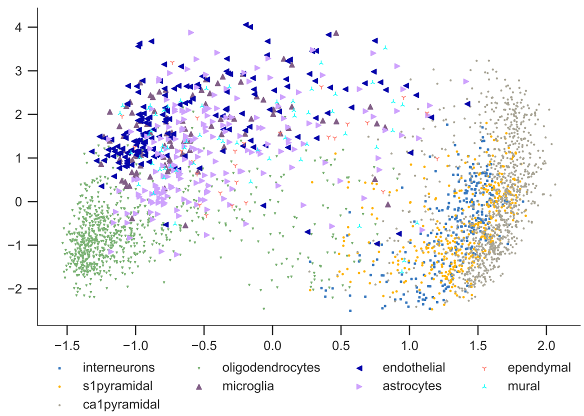

t-SNE	SIMLR
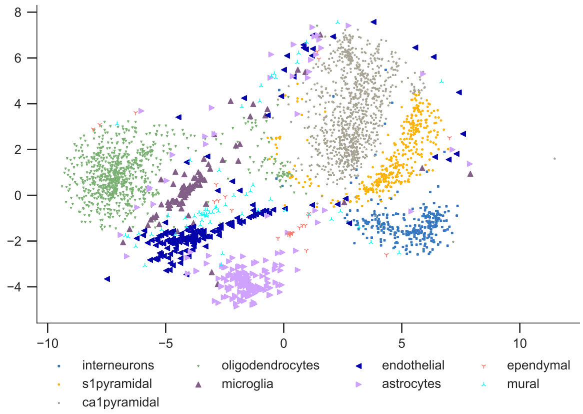	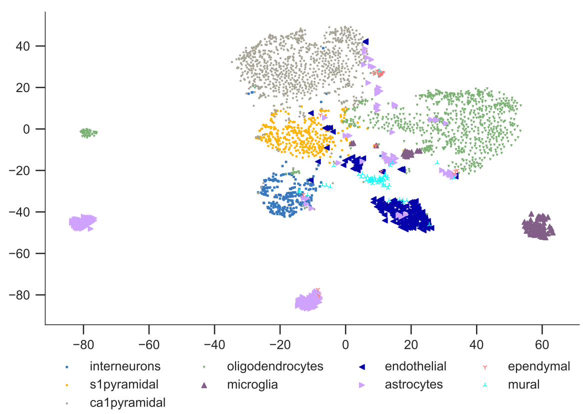

VASC	Clustering
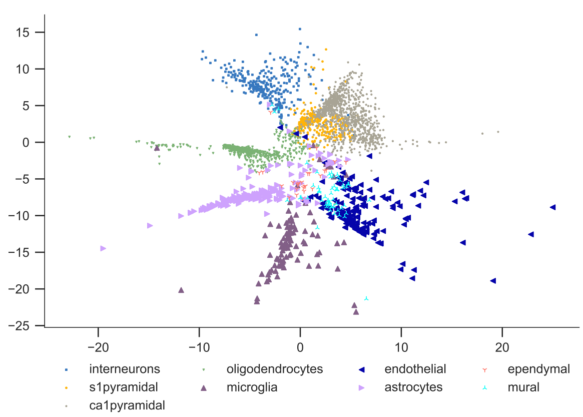	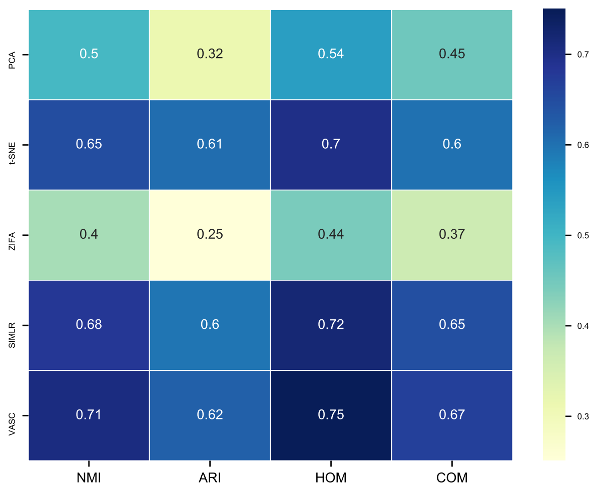

5 Iteration process

We used the Kolodziejczyk dataset to visualize our training procedure. This dataset is composed of three cell types called 2i, a2i, and lif (serum), and cells were obtained by different batches. We showed the first two dimension results of this dataset at epoch 0, 10, 100, and the convergent results (epoch 301) in the following figure. We marked the cell types using different shapes and batches using different colors. As we expected, initially, all kinds of cells tend to clump. Just after 10 epochs, different kinds of cells begin to split, but the batches are still mixed. After 100 epochs, these batches begin to split, too. And finally, all batches are almost distributed in different regions, but we still see that the same type of cells tend to be clustered closer. The shape at epoch 10 using VASC looks like that obtained using PCA. And then, the iteration may mainly be dominated by the secondary variance, such as the batches.

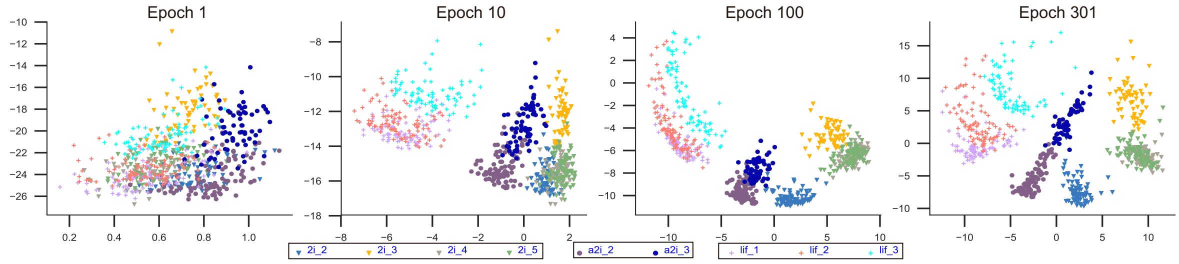

6 Reproducibility of VASC and the zero-inflated layer

We ran our methods for 20 times in some datasets with a small number of cells and golden cell type labels: Biase, Goolam, Pollen, and Yan. We observed consistent results in terms of NMI, ARI, COM, and HOM values. VASC with the zero-inflated layer produced better results than that without the zero-inflated layer.

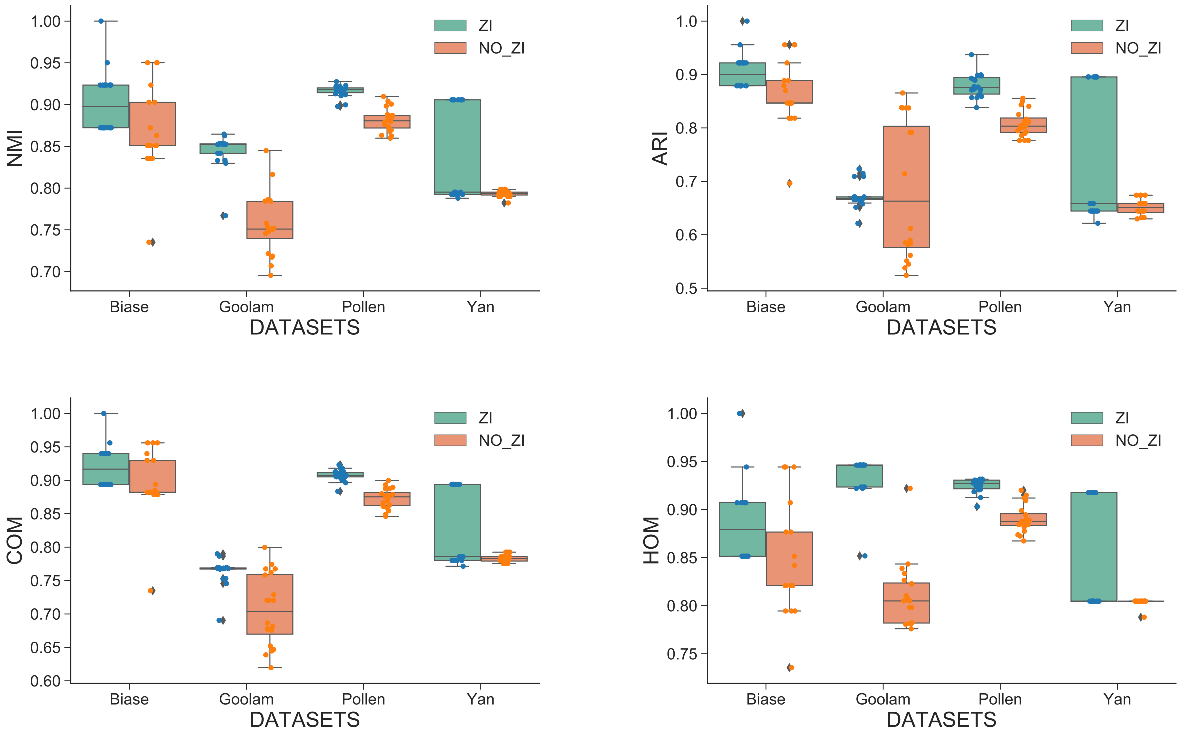
NMI
ARI
COM
HOM
ZI
NO_ZI
Biase
Goolam
Pollen
Yan
DATASETS
